# Top-Down Proteoform Analysis by 2D MS with Quadrupolar Detection

**DOI:** 10.1021/acs.analchem.3c02225

**Published:** 2023-10-25

**Authors:** Marek Polák, Michael Palasser, Alan Kádek, Daniel Kavan, Christopher A. Wootton, Marc-André Delsuc, Kathrin Breuker, Petr Novák, Maria A. van Agthoven

**Affiliations:** † Institute of Microbiology of the Czech Academy of Sciences, Prague 14220, Czech Republic; ‡ Faculty of Science, Charles University, Prague 12843, Czech Republic; § Center for Chemistry and Biomedicine, University of Innsbruck, Innrain 80/82, 6020 Innsbruck, Austria; ∥ Bruker Daltonics GmbH & Co KG, Fahrenheitstraße 4, 28359 Bremen, Germany; ⊥ Institut de Génétique et de Biologie Moléculaire et Cellulaire, INSERM, U596, CNRS, UMR7104, Université de Strasbourg, 1 rue Laurent Fries, 67404 Illkirch-Graffenstaden, France

## Abstract

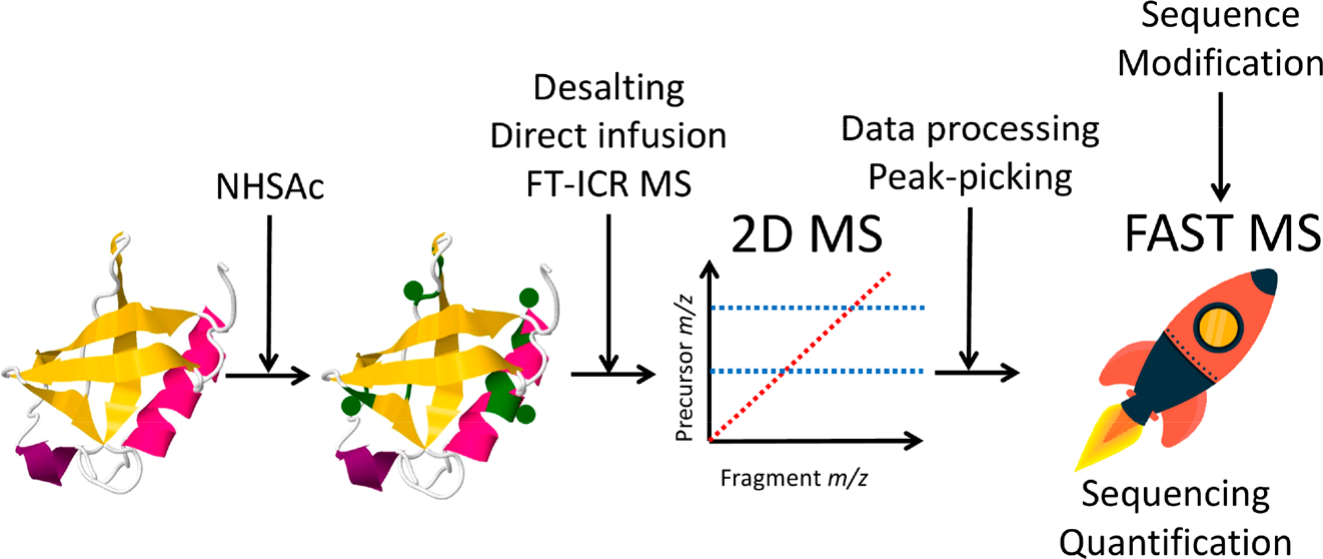

Two-dimensional mass spectrometry (2D MS) is a multiplexed tandem mass spectrometry method that does not rely on ion isolation to correlate the precursor and fragment ions. On a Fourier transform ion cyclotron resonance mass spectrometer (FT-ICR MS), 2D MS instead uses the modulation of precursor ion radii inside the ICR cell before fragmentation and yields 2D mass spectra that show the fragmentation patterns of all the analytes. In this study, we perform 2D MS for the first time with quadrupolar detection in a dynamically harmonized ICR cell. We discuss the advantages of quadrupolar detection in 2D MS and how we adapted existing data processing techniques for accurate frequency-to-mass conversion. We apply 2D MS with quadrupolar detection to the top-down analysis of covalently labeled ubiquitin with ECD fragmentation, and we develop a workflow for label-free relative quantification of biomolecule isoforms in 2D MS.

## Introduction

In mass spectrometry (MS)-based structural analysis of biomolecules, there are multiple methods available to probe three-dimensional structures: noncovalent labeling such as hydrogen–deuterium exchange, radical or oxidative foot-printing (for example, fast photochemical oxidation of proteins), amino acid- or base-selective probes, and chemical cross-linking.^1−6^ The MS analysis of labeled biomolecules is performed by either a bottom-up or a top-down approach.^7−9^

High-resolution mass analyzers such as the Orbitrap or Fourier transform ion cyclotron resonance mass spectrometers (FT-ICR MS) enable the top-down tandem mass analysis of large biomolecules with complex fragmentation patterns.^10^ The development of fragmentation methods that result in high sequence coverage and favor backbone fragmentation, such as electron capture dissociation (ECD) or ultraviolet photodissociation (UVPD), increases the accuracy of the location of the modifications induced by the chemical probing method.^11,12^ Choosing top-down over bottom-up analysis reduces the number of experimental steps and the risk of losing the labels introduced by the probing methods.^13^

Nevertheless, top-down analysis comes with its own set of limitations. Because of the complexity and number of accessible dissociation pathways, ECD and UVPD often yield low-abundance fragments. As a result, they usually require the accumulation of approximately 10–100 measurements to obtain a satisfactory signal-to-noise ratio (SNR).^14,15^ ECD and UVPD are therefore difficult fragmentation methods to couple with liquid chromatography (LC), which does not allow for the accumulation of much more than 10 scans for each analyte because of the rate of change of elution profile, even when using very fast and relatively low-resolution individual measurements.^12^ In addition, standard tandem mass spectrometry techniques require the isolation of a single ion species to enable correlation between the precursor and fragment ions, most often with a quadrupole mass filter.^16^ This method of isolation creates a competition between the accuracy of the isolation and precursor ion abundances. The method also depends on the analytes of interest, thereby making data-independent acquisition difficult.^17,18^ Moreover, for the analysis of protein modifications, no quadrupole-based isolation can separate overlapping isotopic distributions, although adding an ion mobility step has shown advantages.^19,20^ Separation between isobaric ion species and coeluting species is therefore a limitation that all existing data-independent acquisition methods have in common.^21^

Two-dimensional mass spectrometry (2D MS) is a data-independent method for tandem mass spectrometry that does not require ion isolation or separation before fragmentation to correlate between precursor and fragment ions.^22^ In a 2D FT-ICR MS experiment, ion radii are modulated in the ICR cell according to their cyclotron frequencies (which are inversely proportional to their mass-to-charge ratios, or *m*/*z*) before fragmentation with a radius-dependent fragmentation method such as infrared multiphoton dissociation (IRMPD), ECD, or UVPD.^23,24^ The resulting fragment ion abundances (and therefore intensities) are modulated according to the cyclotron frequencies of the precursor ions.^25^ The data set acquired in 2D MS experiments can be Fourier transformed to yield a two-dimensional mass spectrum (2D mass spectrum) that shows the fragmentation pattern of each precursor ion species analyzed in the ICR cell.^24^

2D MS has been applied to the analysis of small molecules, agrochemicals, polymers, and protein tryptic digests and the top-down analysis of proteins.^26−29^ 2D MS has also been used for the label-free relative quantification of modified peptides in a proof-of-concept study.^30−32^ One application of label-free quantification by 2D MS is the top-down analysis of covalently labeled proteins.

New developments in ICR cells have enabled increased resolving power and SNR in FT-ICR MS, which have improved top-down approaches for protein footprinting techniques.^7,33^ In mass spectrometers equipped with dynamically harmonized ICR cells, quadrupolar 2ω detection can be optimized with the appropriate electronics. By detecting ion signals at the 2ω harmonic, the resolving power can be doubled for a given transient length or the transient length can be halved for a given resolving power-.^34^ In this study, we perform 2D MS for the first time on a dynamically harmonized ICR cell with quadrupolar detection to determine the protein’s solvent-accessible surface area. We then compare our results with a previously published study performed using standard tandem mass spectrometry on FT-ICR MS by isolating the [M + 10H]^10+^ charge state of ubiquitin with increasing concentration of an acetylation reagent and fragmenting the ions by collision-induced dissociation (CID).^35^

In this study, we discuss the benefits of quadrupolar 2ω detection in 2D MS and our adapted data processing pipelines for the analysis of different proteoforms. We acetylated ubiquitin with a fivefold molar excess of *N*-hydroxysuccinimidyl acetate (NHSAc), and reaction products were analyzed with top-down 2D MS with ECD fragmentation. We show how 2D MS can be used for the analysis of the covalently labeled protein and what analytical information can be gleaned from 2D MS that cannot be obtained by isolating precursor ions before fragmentation.

## Experimental Methods

### Sample Preparation

The acetylation of ubiquitin (50 μg) was achieved by diluting the sample in 50 mM triethylamine/bicarbonate (pH 7.6, Sigma-Aldrich, Saint Louis, MO) buffer at 0.5 mg/mL and adding the solution to a fivefold molar excess of NHSAc (Tokyo Chemical Industry Co Ltd., Tokyo, Japan) at room temperature for 1 h. The sample was desalted on an OPTI-TRAP macrotrap column (Optimize Technologies, Oregon City, OR) using an aqueous solution with 0.1% formic acid and eluted using an 80% acetonitrile/20% water solution with 0.1% formic acid. The solution was diluted to a 2 μM final protein concentration in aqueous solution of 1% acetic acid and 50% methanol for analysis (all solvents were LC-MS grade and obtained from Merck, Darmstadt, Germany).

### Instrument Parameters

All experiments were performed on a 12 T solariX FT-ICR mass spectrometer (Bruker Daltonik, Bremen, Germany) with an electrospray ion source operated in positive mode and direct infusion at a flow rate of 108 μL/h.^36^ Ions were accumulated for 0.5 s before being transferred to the dynamically harmonized ICR cell (2XR Paracell). The one-dimensional mass spectrum was acquired over an *m*/*z* range of 196.51–3000 in quadrupolar detection mode at the 2ω harmonic as described by Nikolaev *et al.*, with a 1 M data point transient with 64 averaged scans.^37,38^

**Scheme 1:**
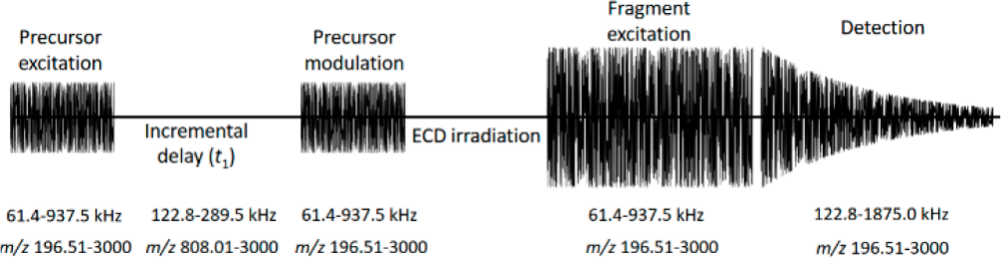
Pulse Sequence for the 2D MS Experiment with Frequency and *m/z* Range for Quadrupolar Detection Dimensions are not to scale.

The pulse sequence for the 2D MS experiment is shown in Scheme 1. The two pulses in the encoding sequence (precursor detection and modulation) were set at 5.02 dB attenuation with 1.0 μs per excitation frequency step (frequency decrements were 625 Hz). The corresponding amplitude was estimated at 250 V_pp_, with a 1.9% sweep excitation power for an amplifier with a maximum output of 446 V_pp_. The encoding delay *t*_1_ was increased 4096 times with a 3 μs increment, which corresponds to a 166.67 kHz frequency range. No phase-cycled signal averaging was employed in the experiment. Because of the digital clock in the Bruker electronics in quadrupolar 2ω detection, the minimum cyclotron frequency for the modulated precursor ions was 122.8 kHz for a maximum *m*/*z* of 3000 during excitation, leading to a *m*/*z* 808.1–3000 mass range for precursor ions. Captured ions were fragmented by ECD using the following parameters: the hollow cathode current was 1.3 A, the ECD pulse length 10 ms, the ECD lens 7 V, and the ECD bias 1.0 V.^39^ Finally, in the horizontal fragment ion dimension, the excitation pulse in the detection sequence was set at 2.60 dB attenuation with a 15 μs/frequency step (frequency decrements were 625 Hz). The corresponding amplitude was estimated at 330 V_pp_, with a 37% sweep excitation power for an amplifier with a maximum output of 446 V_pp_. The horizontal mass range was *m*/*z* 196.51–3000 (corresponding to a frequency range of 1875.0–122.8 kHz). Transients were acquired over 0.559 s with 1 million data points. The total duration of the experiment was 68 min.

### Data Processing

The two-dimensional mass spectrum was processed and visualized using the Spectrometry Processing Innovative Kernel (SPIKE) software (available at www.github.com/spike-project, version 0.99.27, accessed on June 1, 2021) developed by the University of Strasbourg (Strasbourg, France) and CASC4DE (Illkirch-Graffenstaden, France) in the 64-bit Python 3.7 programming language on an open-source platform distributed by the Python Software Foundation (Beaverton, OR).^40^ Processed data files were saved using the HDF5 file format. The 2D mass spectrum was apodized with the Kaiser apodization, zerofilled once, denoised with the SANE algorithm (with a rank of 30), and visualized in magnitude mode.^41^ The size of the resulting data sets was 1 048 576 data points horizontally (fragment ion dimension) by 4096 data points vertically (precursor ion dimension).

Frequency-to-mass conversion was quadratic in both the vertical precursor ion dimension and the horizontal fragment ion dimension.^42^ However, due to the quadrupolar 2ω detection, the parameters of the conversion equation were specific to each dimension, as will be discussed in the next section.^34^ For each precursor ion species, five fragment ion scans were added up to cover the entire precursor isotopic distribution and obtain complete isotopic distributions for all fragment ions. The resulting one-dimensional fragment ion patterns were peak-picked in SPIKE. Peak assignments were performed using the Free Analysis Software for Top-down Mass Spectrometry (FAST-MS) developed by the University of Innsbruck (Innsbruck, Austria) in the 64-bit Python 3.7 programming language.^43^ FAST-MS generated theoretical *c*/*z* and *y* fragment lists for ubiquitin variably modified with 4–6 acetylations located on lysine and methionine residues.

## Results and Discussion

In this study, the 2D MS experiment is performed in a dynamically harmonized ICR cell with quadrupolar 2ω four-plate detection.^34,44^ The ICR cell was “shimmed” to ensure that the precursor ions were centered at the start of the pulse sequence (see Scheme 1).^38^ The frequency range of the broadband pulses for precursor ion excitation and modulation covers the reduced cyclotron frequencies of the precursor and fragment ions (61.4–937.5 kHz). The frequencies measured during the transient cover the second harmonic of the reduced cyclotron frequencies of the precursor and fragment ions (122.8–1875.0 kHz). In addition, the digital modulation frequency was set by the instrument at twice the frequency of the highest *m*/*z* in the excitation pulse, instead of its cyclotron frequency as in detection of the fundamental frequencies.^24^

The first consequence of using quadrupolar detection is that, for an equivalent resolution and *m*/*z* range, each transient duration is halved, resulting in 2D MS experiments that are less time- and sample-consuming. The resolving power in the horizontal fragment ion dimension remains theoretically unchanged, while the SNR in quadrupolar 2ω detection is typically reduced compared to that in standard detection.^45,46^ Second, the coefficients required in the frequency-to-mass conversion equation of 2D mass spectra recorded with quadrupolar 2ω detection are doubled in the horizontal fragment ion dimension compared to the coefficients for the frequency-to-mass conversion in the vertical fragment ion dimension. Finally, the digital modulation frequency set by the instrument electronics is doubled in quadrupolar 2ω detection compared to that in the detection of the fundamental frequencies (see Scheme 1).The modulation frequency for a precursor ion is defined as *f*_ICR_ – *f*_min_, where *f*_ICR_ is the reduced cyclotron frequency of the ion and *f*_min_ is the digital modulation frequency set by the instrument electronics. Doubling *f*_min_ increases the lowest precursor *m*/*z*, which corresponds to a cyclotron frequency of *f*_N_ + *f*_min_, where *f*_N_ is the Nyquist frequency or reduces the necessary Nyquist frequency.^22^ In the 2D MS experiment, the Nyquist frequency in the vertical dimension corresponds to the cyclotron frequency range of the precursor ions. With all other parameters remaining equal, reducing the frequency range increases the theoretical resolving power of the 2D mass spectrum in the vertical dimension.^30^

**Figure 1:**
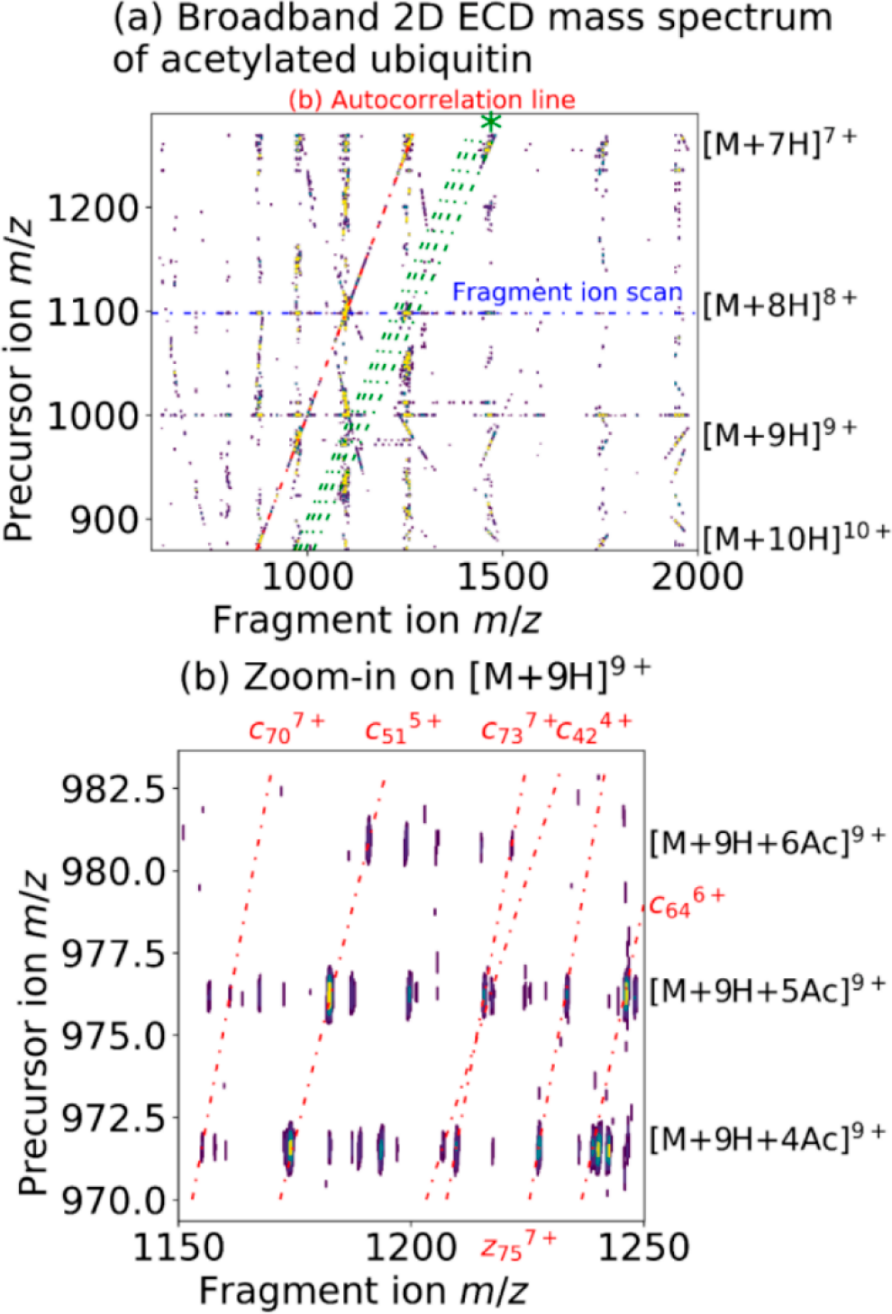
(a) 2D ECD mass spectrum of acetylated ubiquitin. An asterisk (*) indicates electron capture lines (green). (b) Zoom-in on the fragmentation pattern of [M + H]^9+^ with 4–6 acetylations. The red lines indicate dissociation lines for the various *c* and *z* fragments listed around the periphery.

Figure 1a displays the 2D ECD mass spectrum of acetylated ubiquitin. Fragment *m*/*z* values are plotted horizontally, and precursor *m*/*z* values are plotted vertically. The autocorrelation line (*m*/*z*)_precursor_ = (*m*/*z*)_fragment_ (i.e., identity line) results from the modulation of precursor ion radii and abundances with their own reduced cyclotron frequencies and shows all the precursor ions observed in the 2D MS analysis. Horizontally, fragment ion scans show the fragmentation pattern of each precursor ion. Vertically, precursor ion scans show all the precursors of a given fragment ion. The horizontal resolving power (*m*/Δ*m*, where Δ*m* is the full-width at half-maximum of the fragment ion peak) was measured to be 200 000 at *m*/*z* 400 and the vertical resolving power was 1300 at *m*/*z* 874 (corresponding to 2800 at *m*/*z* 400). We can also extract electron capture lines as follows:
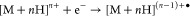
1
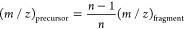
2where *n* is the charge state of the precursor ions. In Figure 1a, electron capture lines for the capture of one electron by the 7–10+ charge states are plotted in green. As shown in eq 2, their slopes are 6/7, 7/8, 8/9, and 9/10.

The 2D ECD mass spectrum also shows harmonics of the autocorrelation line as curved lines. The presence of harmonic peaks is caused by the nonsinusoidal modulation of the precursor ions.^22,25^ Scintillation noise, which is caused by the fluctuation of the number of ions in the ICR cell from scan to scan, manifests as vertical streaks along the *m*/*z* of the precursor ions and can be filtered out by the use of a denoising algorithm during data processing.^41^Figure S1 in the Supporting Information shows the complete 2D mass spectrum, including harmonics of the autocorrelation line. Most harmonics are similar to the ones obtained in 2D MS with standard detection at 1ω. One noticeable difference between detection at 1ω and quadrupolar detection at 2ω is the presence of the 1ω subharmonic frequency (at double the measured *m*/*z*). In the 2D mass spectrum, we observe the subharmonic peak of the autocorrelation line at a 1/2 slope at approximately 15–20% the intensity of the autocorrelation line.^24^

Here, the 2D mass spectrum is shown as a contour plot, but we cannot see enough detail to show the fragmentation patterns of the 7–10+ charge states of acetylated ubiquitin. Because of the multiplicity of dissociation channels for the fragmentation of proteins in ECD, relative intensities of fragment ions in the 2D mass spectrum can be equivalent to the intensity of signals caused by harmonics or noise, and plotting one without the other is difficult.^47^ Nevertheless, discriminating analytically useful signal from noise is readily achieved because, due to distinctly different frequency relationships, they are in different areas of the spectrum. The zoomed-in view of the fragmentation patterns shown in Figure 1b illustrates how the fragmentation patterns can be easily distinguished. The red lines highlight various dissociation lines to illustrate how they can be used to locate modifications.

**Figure 2:**
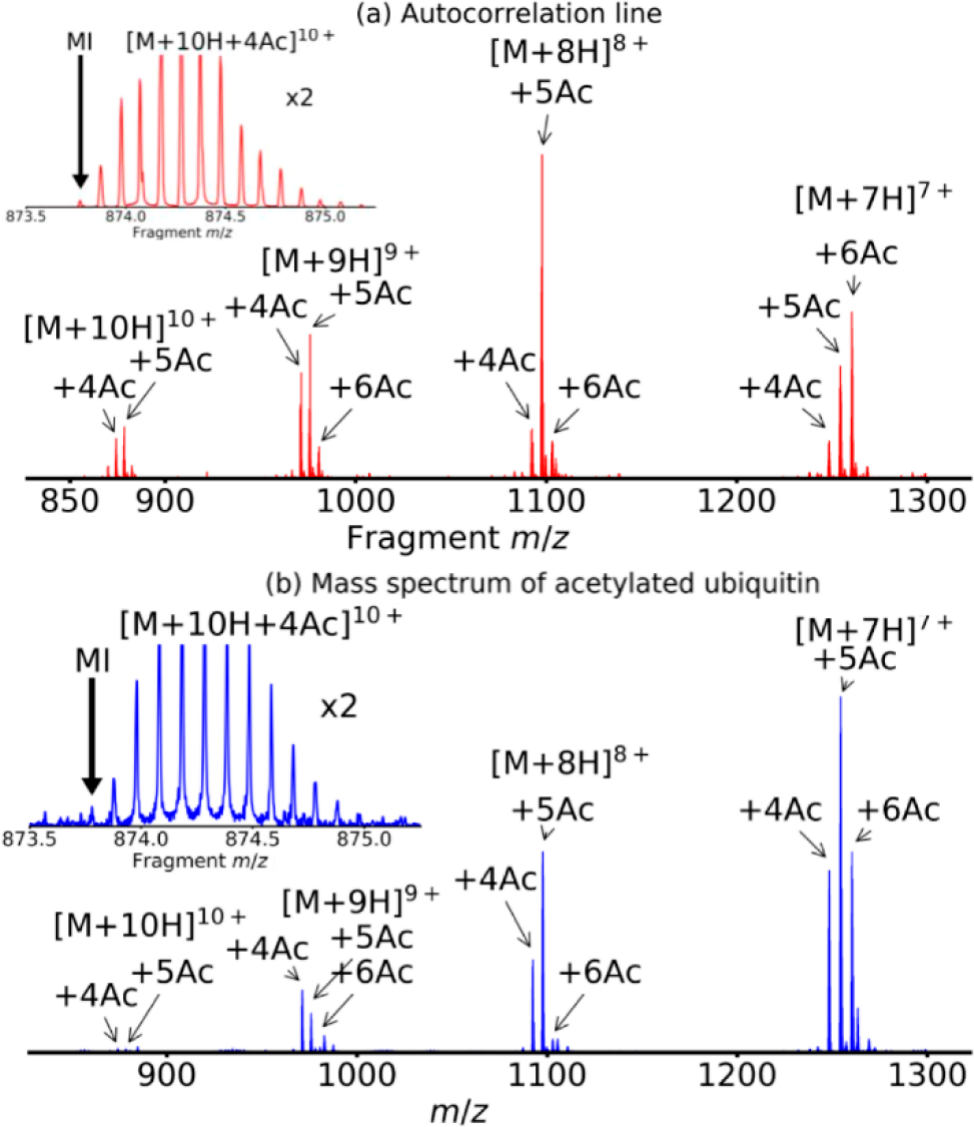
(a) Extracted autocorrelation line from the 2D mass spectrum. The inset shows a zoomed-in view of the isotopic distribution of the [M + 10H + 4Ac]^10+^. The arrow marks the monoisotopic peak (MI). (b) Mass spectrum of acetylated ubiquitin. The inset shows the zoomed-in isotopic distribution of the [M + 10H + 4Ac]^10+^ species from the mass spectrum shown in Figure 2b. The arrow marks the monoisotopic peak (MI).

Figure 2a shows the extracted autocorrelation line (*m*/*z* 850–1300) of the 2D ECD mass spectrum. The charge states of acetylated ubiquitin that are modulated and fragmented in this 2D mass spectrum are 7–10+, each of them bearing 4–6 acetylations, which is consistent with the level of acetylation under similar labeling conditions presented by Novák *et al*.^35^ The inset shows the isotopic distribution of the [M + 10H + 4Ac]^10+^ precursor ion species on the autocorrelation line. The signal from precursor ions is modulated by the radius (during the pulse-delay-pulse sequence in Scheme 1) and by their abundance (during the ECD irradiation), followed by Fourier transformation over 4096 scans. Therefore, the SNR on the autocorrelation line is typically very high.^48^ In the case of the isotopic distribution of [M + 10H + 4Ac]^10+^, the SNR for the most intense peak is 720. The SNR for the monoisotopic peak is 20. For comparison, Figure 2b shows the 1D mass spectrum of acetylated ubiquitin. Both the mass spectrum and the autocorrelation line show similar charge state ranges and acetylation numbers for each charge state. However, the relative intensities of the peaks are different between Figure 2a and Figure 2b: while the relative intensities in the mass spectrum reflect ion abundance and charge state, the relative intensities on the autocorrelation line also reflect the fragmentation efficiency of each ion species, which, for ECD, depends greatly on charge state.^24,49^ The SNR for the monoisotopic peak of [M + 10H + 4Ac]^10+^ in the mass spectrum is only 2–3, which is about 10× smaller than that for the same monoisotopic peak extracted from the autocorrelation line in Figure 2a. With 4096 scans instead of 64, the SNR would be 8× higher.

**Figure 3:**
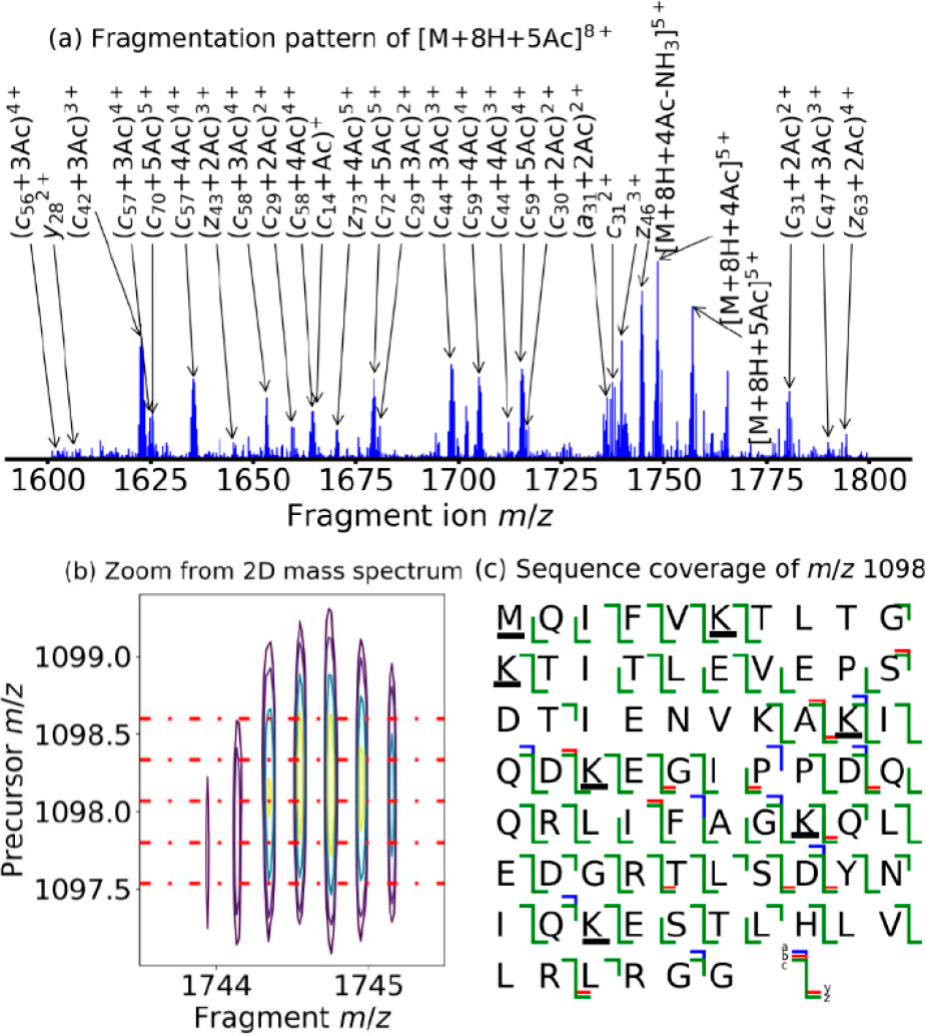
(a) Added-up fragment ion scans of *m*/*z* 1098 ([M + 8H + 5Ac]^8+^) extracted from the 2D mass spectrum of acetylated ubiquitin. (b) Zoom-in on the isotopic distribution of [M + 8H + 4Ac – NH_3_]^5+^ from the [M + 8H + 5Ac]^8+^ precursor, with lines marking the fragment ion scans added up to obtain Figure 2a. (c) Sequence coverage of [M + 8H + 5Ac]^8+^, totaling 86% (acetylated residues underlined).

One issue in the top-down analysis of large biomolecules is their accurate mass determination. Typically, deconvolution algorithms based on the averagine method are used because the SNR of the monoisotopic peak is often below the level of detection.^50^ Although most biomolecules for which this issue arises are much larger than ubiquitin, this result suggests that using the autocorrelation line in 2D mass spectra may offer more accurate analytical information by offering higher SNRs for monoisotopic peaks of biomolecules. The process of peak assignment and sequence coverage determination using FAST-MS is illustrated in Figure 3 for each ubiquitin isoform. Figure 3a shows the summed fragment ion scans of *m*/*z* 1098 ([M + 8H + 5Ac]^8+^). Five fragment ion scans were extracted from the 2D mass spectrum to cover the precursor ion peak of [M + 8H + 5Ac]^8+^ at *m*/*z* 1098 and co-added to obtain the resulting fragment ion scan shown in Figure 3a. In Figure 3b, we illustrate why the fragment ion scans were added up (individual extracted scans are shown in red). Since the resolving power in the vertical precursor ion dimension is insufficient to distinguish between precursor ion isotopes, the overlap between precursor ion isotopic peaks is not complete. The relative intensities in fragment ion isotopic distributions in a single fragment ion scan can therefore be distorted; to recover the full isotopic distribution for fragment ions, we summed up the fragment ion scans before analysis. FAST-MS compares experimental and theoretical relative intensities to gauge the quality of peak assignments, peak-picking the fragmentation pattern for the full isotopic distribution of each protein isoform, then improves the accuracy of the sequence coverage assignment, which provides an optional advantage of adding-up adjacent scans in 2D MS. Because the fragment ion scans are adjacent, noise signals are correlated between them and the SNR is only marginally affected.

The information fed into FAST-MS was the ubiquitin sequence, the molecular formula of the acetylation, the number of modifications, and the location of the modification (M and K residues). The software then generated a library of theoretical isotopic distributions of the *a*, *b*, *c*, *y*, and *z* fragments. Figure 3c shows the sequence coverage of [M + 8H + 5Ac]^8+^. All peak assignments were validated manually, reaching a sequence coverage of 86%. For comparison, a one-dimensional tandem mass spectrum of [M + 8H + (0–6)Ac]^8+^ in similar conditions with 2 M data points and 200 accumulated scans yielded a cleavage coverage of 84% (see Table S12 and Figure S2 in the Supporting Information).

The lists of peak assignments can be found in Tables S1–S11 in the Supporting Information. Table 1 summarizes the sequence coverage for each proteoform and charge state of acetylated ubiquitin. Each fragmentation pattern has a different sequence coverage, which depends on both the abundance of each precursor ion and charge state because the fragmentation efficiency of ECD is charge state-dependent.^11^ The last column shows the sequence coverage for each ubiquitin proteoform after the results for all charge states. Because different fragments are produced for each charge state, the total sequence coverage is higher than the sequence coverage of each charge state.

**Table 1:** Sequence Coverage of Each Precursor Ion in the 2D Mass Spectrum of Acetylated Ubiquitin^a^

	[M + 10H]^10+^	[M + 9H]^9+^	[M + 8H]^8+^	[M + 7H]^7+^	total
+4Ac	34%	63%	36%	16%	84%
+5Ac	57%	67%	86%	46%	89%
+6Ac	N/A	32%	30%	34%	60%

a Legend: Ac = acetylation, N/A = not annotated.

**Figure 4:**
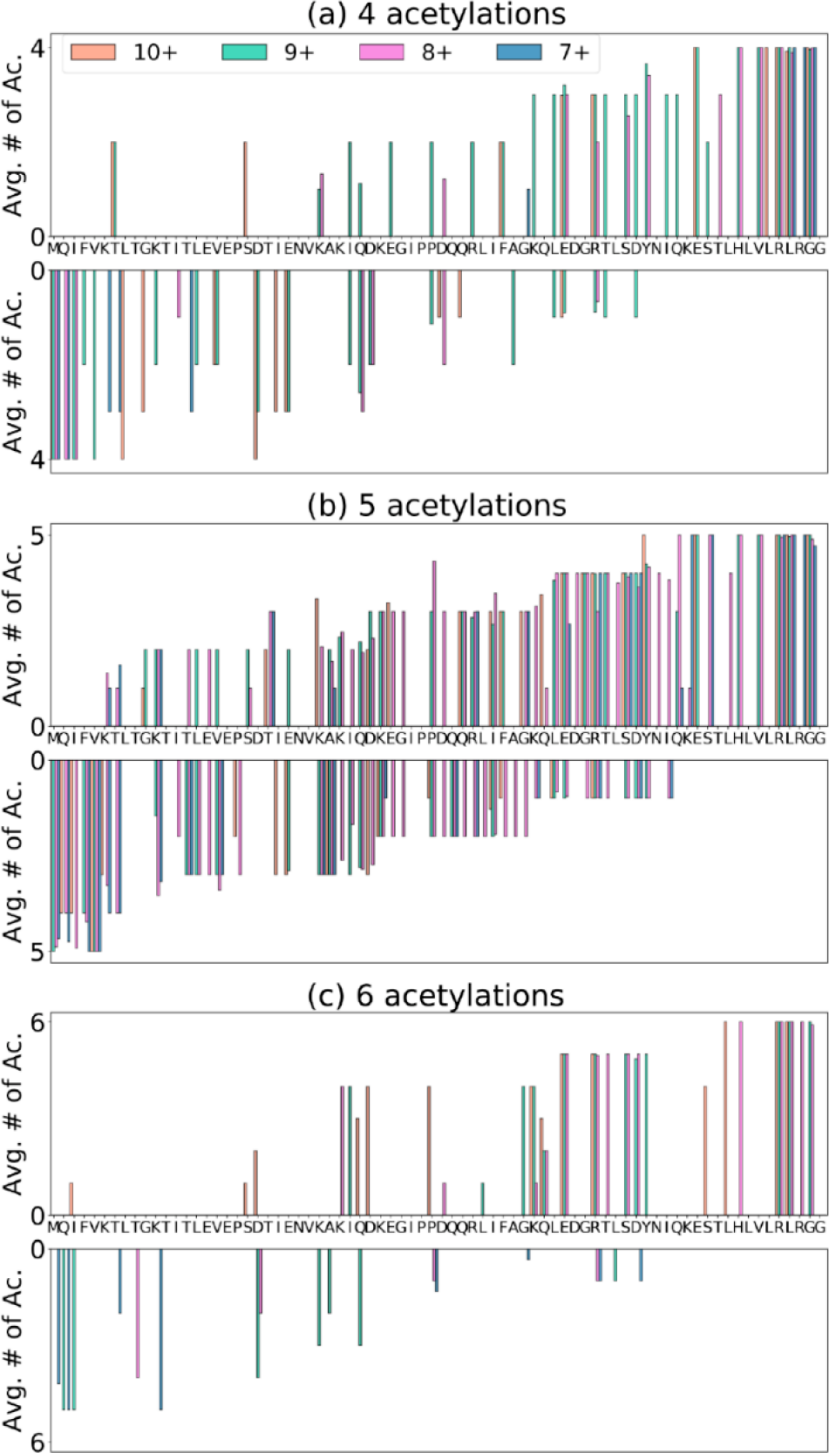
Acetylation rate vs residue index for ubiquitin modified with (a) four acetylations (*c* fragments on top, *z* fragments at the bottom), (b) five acetylations (*c* fragments on top, *z* fragments at the bottom), and (c) six acetylations (*c* fragments on top, *z* fragments at the bottom).

Figure 4 shows the acetylation rate vs the residue index for proteoforms with four, five, and six acetylations, for *c* and *z* fragments. Each plot combines the peak assignments for all charge states (7–10+) with M/K acetylation sites assigned by FAST-MS. These plots allow us to locate acetylation sites and quantify the extent of acetylation.^30−32^

Figure 4a shows the extent of acetylation for ubiquitin with four acetylations from *c* fragments and *z* fragment ions, respectively. Ubiquitin has eight possible acetylation sites, namely, M1, K6, K11, K27, K29, K33, K48, and K63. From the N-terminus, the acetylation sites are M1, K6, K48, and K63. From the C-terminus, the acetylation sites are K63, K48, K33, and K6. The most easily accessible sites can therefore be located at K63, K48, and K6. Residues M1, K11, K27, K29, and K33 are less solvent-accessible. The sequence coverage for ubiquitin with four acetylations is not sufficient to distinguish between K27, K29, and K33.

Figure 4b shows the acetylation rate for ubiquitin with five acetylations from *c* and *z* fragments, respectively. From the N-terminus, the acetylation sites are M1, K6, K29/33, K48, and K63. From the C-terminus, the acetylation sites are K63, K48, K33, K11, and K6 or M1. Figure 4c shows the acetylation rate for ubiquitin with six acetylations from *c* fragments and *z* fragments, respectively. From the N-terminus, the acetylation sites are M1, K6, K11, K29, K48, and K63. From the C-terminus, the acetylation sites are K63, K48, K33, K27, K11, and M1.

From these results, we can conclude that the most accessible acetylation sites are K63 and K48; followed by K6, M1, and K33; and finally K29, K27, and K11. This conclusion is congruent with the conclusions by top-down CID MS/MS found by Novák *et al*.^35^ We should note that we observe a loss of acetylation in Figure 3a. However, despite this result, all acetylation sites for each isoform could be accounted for.

One advantage of broadband-mode 2D MS over individual MS/MS spectra is the ease with which the interactions between the charge state and protein modifications can be measured. Since lysine, which is the main residue carrying the acetylation, also carries the charge, and since acetylation is known for reducing positive charges in proteins, we hypothesized that the charge state of ubiquitin would be affected by acetylation.^51^ We calculated the average charge state of ubiquitin for each number of acetylations using the intensities on the autocorrelation line and the mass spectrum.

Since measured intensities in FT-ICR MS are proportional to the abundance and the charge of each ion species, we calculated the average charge state for each proteoform using the following equation:
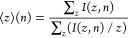
3where ⟨*z*⟩(*n*) is the average charge state for *n* acetylations and *I*(*z*, *n*) is the intensity of the [M + *z*H + *n*Ac]^*z*+^ peaks.

The results are plotted in figure S3 in the Supporting Information. The average charge state decreases with the number of acetylations, both in the mass spectrum and in the autocorrelation line, which is consistent with acetylation reducing the number of positive charges on a protein. The results also show that the average charge state is higher in the autocorrelation line than in the mass spectrum, which is due to factors determining the intensity of a peak in FT-ICR MS. In the mass spectrum, peak intensities are determined by the ion abundance and the charge state. On the autocorrelation line of a 2D ECD mass spectrum, peak intensities are determined by the ion abundance, the charge state and the capacity to capture electrons, which increases with charge state in positive ionization mode.^24^ Therefore, the average charge state for each isoform is higher in the autocorrelation line of the 2D mass spectrum than in the 1D mass spectrum.

**Figure 5:**
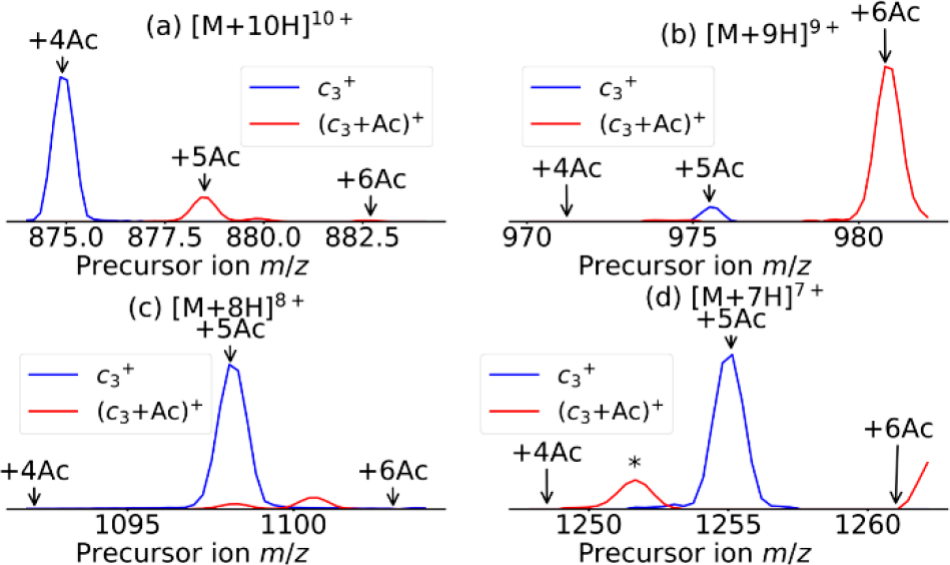
Precursor ion scans for *c*_3_ (*m*/*z* 390.21790, blue) and *c*_3_ + Ac (*m*/*z* 432.22714, red) fragments for (a) 10+, (b) 9+, (c) 8+, and (d) 7+ precursor charge states. An asterisk (*) indicates an artifact caused by the denoising algorithm.

In Figure 5, we seek to determine whether the acetylation of both lysine and N-terminus methionine reduces the charge state of ubiquitin. Therefore, we extracted the vertical precursor ion scans from the 2D mass spectrum for the *c*_3_ (*m*/*z* 390.21790, blue) and *c*_3_+Ac (*m*/*z* 432.22714, red) fragments, which, in turn, enables us to quantify the acetylation of only the M1 residue in ubiquitin. Figure 5 shows the *c*_3_ fragment ion (blue) alongside with its acetylated form (*c*_3_ + Ac, red) for charge states 10–7+ in Figure 5a–d, respectively.

Figure 5a shows that ubiquitin with four acetylations produces the *c*_3_ fragment and that ubiquitin with five and six acetylations produce the *c*_3_ + Ac fragment in the 10+ charge state. Therefore, the fifth most favored acetylation site is M1. In Figure 5b, for 9+ charged precursors, the *c*_3_ + Ac fragment is only produced from the ubiquitin with six acetylations, which means that M1 is the sixth most favored acetylation site. In Figure 5c and d, we see that only *c*_3_ is produced from the 7+ and 8+ charge states, which means that M1 is, at best, the seventh most favored acetylation site. As a result, we can say that ubiquitin with an acetylation on the M1 residue skews toward higher charge states. This result suggests that the acetylation of the methionine residue may not reduce the charge state of ubiquitin like the acetylation of the lysine residues does.

## Conclusion

Stable protein covalent labeling coupled to 2D MS analysis and ECD fragmentation has yielded information about solvent accessibility at individual residues, particularly the N-terminus methionine and the lysines residues.^35^ For the first time, 2D MS was applied with quadrupolar detection on a dynamically harmonized ICR cell. The detection at the 2ω harmonic led to a shorter experimental duration and an increase in resolving power in the vertical precursor ion dimension.^34^

Because of the multiplexing inherent to the 2D MS experiment, we were able to obtain in parallel the ECD fragmentation pattern of four charge states of ubiquitin with up to six acetylations each.^24^ The resolving power in the vertical precursor ion scan was sufficient to confidently correlate precursor and fragment ions without unwanted contributions from different proteoforms and without a loss of precursor ion abundance due to quadrupole isolation. We used the FAST-MS software and defined a workflow to assign all fragment ions generated from each charge state by ECD and quantify the extent of acetylation of methionine/lysine residues, which was consistent with previously published results.^30,43^^35^

2D MS showed the advantages of having the fragmentation patterns of multiple isoforms and charge states in a single spectrum. First, the sequence coverage from the combined fragmentation patterns of all observed charge states was higher than the sequence coverage obtained from the charge state with the highest fragmentation efficiency. Second, the 2D mass spectrum enabled the observation that acetylation reduces the gas-phase charge state of ubiquitin and more specifically that the acetylation of lysine residues reduces the charge state to a higher degree than the acetylation of the N-terminus M1 residue.

This study shows the potential for 2D MS coupled with ECD fragmentation to yield comprehensive analytical information for the top-down analysis of the proteoform mixtures. 2D ECD MS can further be applied to the quantitative analysis of post-translational modifications of proteins and to the structural analysis of covalently labeled proteins.

## Data Availability

Raw data and processing parameter file available at https://zenodo.org/uploads/10027010.

## Supporting Information Available

Peaklists and assignments for the extracted fragment ion scans from the 2D mass spectrum and the tandem mass spectrum, full 2D mass spectrum, and plot of the average charge state of each proteoform (PDF)
