# Supplementary material for: Top-Down Proteoform Analysis by 2D MS with Quadrupolar Detection
Source: Anal Chem. 2023 Oct 25;95(44):16123–30. doi: 10.1021/acs.analchem.3c02225 (PMC10633810; doi:10.1021/acs.analchem.3c02225)
Supplement: Supplementary file 1 — ac3c02225_si_001.pdf [file ac3c02225_si_001.pdf]

Top-down proteoform analysis by 2D MS with quadrupolar detection

Supporting Information

Marek Polák[1,2], Michael Palasser[3], Alan Kádek[1,4,5], Daniel Kavan[1,2], Christopher A. Wootton[6], Marc-André Delsuc[7], Kathrin Breuker[3], Petr Novák[1,2*], Maria A. van Agthoven[1,3*]

[1]Institute of Microbiology of the Czech Academy of Sciences, Prague, 14220, Czech Republic
[2]Faculty of Science, Charles University, Prague, 12843, Czech Republic
[3]Center for Chemistry and Biomedicine, University of Innsbruck, Innrain 80/82, 6020 Innsbruck, Austria
[4]Leibniz Institute of Virology, 20251 Hamburg, Germany
[5]European XFEL GmbH, 22869 Schenefeld, Germany
[6]Bruker Daltonics GmbH & Co KG, Fahrenheitstraße 4, 28359 Bremen, Germany
[7]Institut de Génétique et de Biologie Moléculaire et Cellulaire, INSERM, U596, CNRS, UMR7104, Université de Strasbourg, 1 rue Laurent Fries, 67404, Illkirch-Graffenstaden, France

* Corresponding authors: Petr Novák: pnovak@biomed.cas.cz, Maria van Agthoven: maria.vanagthoven@biomed.cas.cz

Table of Content:

Figure S1. Full-sized 2D ECD mass spectrum of acetylated ubiquitin p.2

Table S1. Peak assignments for the $[M+10H+4Ac]^{10+}$ fragment ion scan (*m/z* 874) p.3

Table S2. Peak assignments for the $[M+10H+5Ac]^{10+}$ fragment ion scan (*m/z* 878) p.4

Table S3. Peak assignments for the $[M+9H+4Ac]^{9+}$ fragment ion scan (*m/z* 972) p.6

Table S4. Peak assignments for the $[M+9H+5Ac]^{9+}$ fragment ion scan (*m/z* 976) p.9

Table S5. Peak assignments for the $[M+9H+6Ac]^{9+}$ fragment ion scan (*m/z* 981) p.12

Table S6. Peak assignments for the $[M+8H+4Ac]^{8+}$ fragment ion scan (*m/z* 1093) p.13

Table S7. Peak assignments for the $[M+8H+5Ac]^{8+}$ fragment ion scan (*m/z* 1098) p.15

Table S8. Peak assignments for the $[M+8H+6Ac]^{8+}$ fragment ion scan (*m/z* 1103) p.20

Table S9. Peak assignments for the $[M+7H+4Ac]^{7+}$ fragment ion scan (*m/z* 1249) p.21

Table S10. Peak assignments for the $[M+7H+5Ac]^{7+}$ fragment ion scan (*m/z* 1255) p.22

Table S11. Peak assignments for the $[M+7H+5Ac]^{7+}$ fragment ion scan (*m/z* 1260) p.24

Figure S2. Tandem mass spectrum of acetylated ubiquitin. p.26

Table S12. Peak assignments for the tandem mass spectrum (*m/z* 1097) p.27

Figure S3. Average charge state vs. number of acetylations p.34

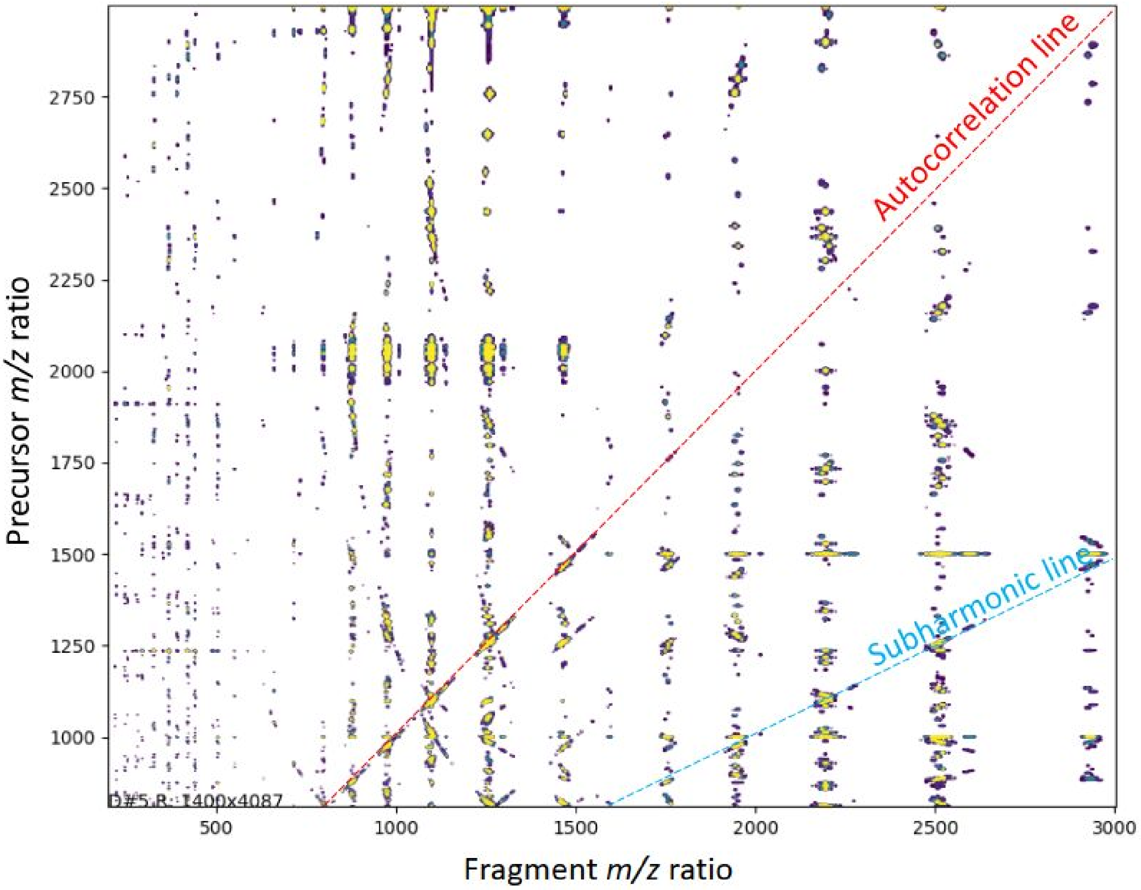

Figure S1. Full-sized 2D ECD mass spectrum of acetylated ubiquitin.

Table S1. Peak assignments for the $[M+10H+4Ac]^{10+}$ fragment ion scan (*m/z* 874).

m/z ratio	*z*	name	Mass accuracy (ppm)	S/N
475.26337	2	$(c_7+2Ac)^{2+}$	2.01	2.80
640.04196	3	$z_{17}{}^{3+}$	0.33	10.32
856.66950	5	$(z_{37}+Ac)^{5+}$	0.00	9.85
869.57103	10	$[M+10H +3Ac]^{10+}$	-1.25	10.07
873.77031	10	$[M+10H +4Ac]^{10+}$	-3.28	23441.85
956.51089	3	$(z_{25}+Ac)^{3+}$	-2.33	19.47
964.30292	9	$(c_{75}+4Ac)^{9+}$	0.84	19.92
966.07931	9	$[M+9H +3Ac]^{9+}$	-0.01	674.41
968.96778	9	$[M+9H+4Ac-NH_3]^{9+\bullet}$	0.49	686.54
970.02177	8	$(z_{68}+4Ac)^{8+\bullet}$	1.87	4.90
970.86620	6	$(c_{51}+2Ac)^{6+\bullet}$	3.89	1.12
970.93254	5	$c_{43}{}^{5+}$	0.00	30.04
978.03310	6	$(c_{51}+3Ac)^{6+}$	0.97	81.82
1000.20716	6	$(z_{52}+3Ac)^{6+}$	-2.74	7.67
1000.37481	6	$(z_{52}+3Ac)^{6+\bullet}$	-3.06	6.11
1011.20178	3	$(c_{26}+3Ac)^{3+}$	0.00	3.38
1021.71685	6	$(z_{53}+3Ac)^{6+}$	-0.14	7.72
1021.88426	6	$(z_{53}+3Ac)^{6+\bullet}$	-0.69	8.47
1032.55958	6	$(c_{54}+3Ac)^{6+\bullet}$	3.69	7.67
1032.72534	6	$(c_{54}+3Ac)^{6+}$	1.55	15.15
1039.56450	5	$(c_{45}+2Ac)^{5+\bullet}$	-1.15	2.24
1039.76501	5	$(c_{45}+2Ac)^{5+}$	-2.16	3.06
1043.94148	8	$(c_{72}+4Ac)^{8+}$	3.20	31.74
1052.69784	8	$(c_{73}+3Ac)^{8+\bullet}$	1.42	4.71
1057.90162	3	$(b_{28}+2Ac)^{3+}$	1.43	3.14
1058.07209	8	$(c_{73}+4Ac)^{8+}$	-1.47	53.64
1061.56183	7	$(c_{64}+4Ac)^{7+}$	-0.62	95.87
1064.40347	6	$(z_{55}+4Ac)^{6+}$	-3.68	5.71
1073.58304	8	$(z_{75}+4Ac)^{8+}$	0.74	143.33
1079.72003	7	$(z_{66}+3Ac)^{7+}$	-3.26	4.36
1084.70839	8	$[M+8H+3Ac-NH_3]^{8+\bullet}$	-2.39	33.28
1100.75184	6	$(y_{57}+4Ac)^{6+}$	-1.66	5.31
1121.76292	6	$(z_{59}+2Ac)^{6+}$	0.00	9.22
1136.62279	2	$(c_{19}+3Ac)^{2+\bullet}$	0.00	9.57
1159.13352	2	$(c_{20}+2Ac)^{2+\bullet}$	0.00	5.21
1170.48385	7	$(c_{71}+4Ac)^{7+\bullet}$	-0.23	7.46
1187.88014	4	$(c_{41}+4Ac)^{4+\bullet}$	3.34	3.57
1280.16676	2	$(b_{22}+3Ac)^{2+}$	0.00	5.89
1342.07865	3	$(z_{35}+Ac)^{3+\bullet}$	3.07	5.23

Average mass accuracy: 1.51 ppm (standard deviation: 1.27 ppm)

Table S2. Peak assignments for the $[M+10H+5Ac]^{10+}$ fragment ion scan (*m/z* 878).

m/z ratio	*z*	name	Mass accuracy (ppm)	S/N
717.91950	2	z_{13}^{2+}	1.09	4.68
801.17745	4	$(c_{27}+4Ac)^{4+}$	-2.60	7.43
809.86904	7	$(c_{49}+4Ac)^{4+}$	-0.14	6.61
839.46013	5	b_{38}^{5+}	-1.49	3.23
847.12881	3	$(z_{22}+Ac)^{3+}$	-1.35	22.04
857.46558	7	$(z_{52}+3Ac)^{7+}$	-1.28	16.70
873.77049	10	$[M+10H+4Ac]^{10+}$	-3.08	32.59
874.22050	4	$(z_{31}+Ac)^{4+}$	-0.97	3.23
875.90467	7	$(z_{53}+3Ac)^{7+}$	3.82	24.71
879.67270	5	$(z_{38}+Ac)^{5+}$	-2.48	12.88
882.07164	5	$(a_{39}+3Ac)^{5+}$	-2.81	6.40
891.33472	7	$(c_{54}+4Ac)^{7+}$	-2.76	11.29
894.98596	6	$(c_{47}+3Ac)^{6+}$	0.57	9.98
915.83264	6	$(a_{48}+4Ac)^{6+}$	-0.17	7.65
918.99944	4	$(c_{32}+2Ac)^{4+}$	-0.25	8.87
921.74349	4	$(z_{32}+2Ac)^{4+\bullet}$	0.47	9.14
929.35876	7	$(z_{57}+2Ac)^{7+}$	3.36	4.68
931.50180	6	$(z_{48}+3Ac)^{6+}$	-0.93	7.07
932.01053	6	$(y_{49}+Ac)^{6+}$	2.23	9.67
934.21767	7	$(c_{57}+4Ac)^{7+\bullet}$	1.14	8.99
934.36090	7	$(c_{57}+4Ac)^{7+}$	0.34	11.66
945.02560	2	$(b_{16}+2Ac)^{2+}$	-0.70	3.19
961.52720	4	$(a_{34}+Ac)^{4+}$	1.20	19.60
962.02810	4	$c_{34}^{4+\bullet}$	1.33	3.54
967.65641	7	$(a_{59}+4Ac)^{7+}$	0.29	20.58
968.85642	9	$[M+9H+4Ac-NH_3]^{9+}$	1.13	87.24
968.85820	9	$(c_{75}+5Ac)^{9+\bullet}$	0.24	70.32
968.96961	9	$(c_{75}+5Ac)^{9+}$	-0.35	85.46
973.63558	9	$[M+9H+5Ac-NH_3]^{9+\bullet}$	0.45	779.67
975.52292	9	$[M+9H+5Ac]^{9+\bullet}$	-4.16	301.73
978.69844	6	$(z_{50}+5Ac)^{6+}$	3.65	7.23
984.86630	6	$(c_{51}+4Ac)^{6+\bullet}$	0.36	24.49
985.03339	6	$(c_{51}+4Ac)^{6+}$	-0.53	65.92
985.20049	6	$(a_{53}+Ac)^{6+}$	0.71	55.01
986.72661	5	$(z_{43}+2Ac)^{5+}$	-1.13	36.23
986.92896	5	$(z_{43}+2Ac)^{5+\bullet}$	-0.34	39.35
987.94935	7	$(b_{60}+4Ac)^{7+}$	2.14	8.22
991.28407	8	$(y_{69}+5Ac)^{8+}$	0.94	13.21
991.40901	8	$(z_{70}+3Ac)^{8+}$	-0.11	14.55
991.53398	8	$(z_{70}+3Ac)^{8+\bullet}$	-1.13	9.83
1000.20875	6	$(z_{52}+3Ac)^{6+}$	-1.15	27.20
1014.79387	4	$(c_{34}+5Ac)^{4+}$	1.86	7.01
1017.92456	8	$(z_{71}+5Ac)^{8+}$	0.91	5.13
1018.75426	5	$(c_{44}+3Ac)^{5+}$	-1.41	21.84
1020.75039	5	$(z_{44}+3Ac)^{5+}$	1.53	16.70

1020.95096	5	$(z_{44}+3\mathrm{Ac})^{5+\bullet}$	0.55	7.20
1035.06543	6	$(a_{55}+2\mathrm{Ac})^{6+}$	5.56	3.26
1039.55683	6	$(c_{54}+4\mathrm{Ac})^{6+\bullet}$	-0.67	8.76
1039.55757	3	$(c_{27}+2\mathrm{Ac})^{3+\bullet}$	-2.95	4.30
1043.44059	8	$(z_{73}+4\mathrm{Ac})^{8+}$	1.13	45.69
1048.17030	5	$(c_{45}+3\mathrm{Ac})^{5+}$	0.89	12.14
1049.18892	8	$(c_{72}+5\mathrm{Ac})^{8+}$	-0.51	76.03
1057.57537	8	$(z_{74}+4\mathrm{Ac})^{8+}$	0.42	50.96
1057.70072	8	$(z_{74}+4\mathrm{Ac})^{8+\bullet}$	-0.17	48.66
1063.32281	8	$(c_{73}+5\mathrm{Ac})^{8+}$	-2.03	89.26
1067.42042	7	$(c_{64}+5\mathrm{Ac})^{7+\bullet}$	0.37	14.18
1067.56365	7	$(a_{65}+4\mathrm{Ac})^{7+}$	-0.33	99.38
1067.90116	3	$(c_{27}+4\mathrm{Ac})^{7+}$	-2.31	4.11
1089.95936	8	$[\mathrm{M}+8\mathrm{H}+4\mathrm{Ac}-\mathrm{NH_3}]^{8+\bullet}$	-2.70	17.09
1143.27002	6	$(c_{59}+5\mathrm{Ac})^{6+}$	0.36	4.73
1177.37597	4	$(c_{41}+3\mathrm{Ac})^{4+\bullet}$	2.07	6.45
1177.62417	4	$(c_{41}+3\mathrm{Ac})^{4+}$	-1.12	14.63
1177.65597	1	$(c_{10}+\mathrm{Ac})^{+\bullet}$	3.06	3.23
1216.05105	5	$(c_{53}+4\mathrm{Ac})^{5+\bullet}$	3.04	2.94
1216.25158	5	$(c_{53}+4\mathrm{Ac})^{5+}$	2.18	3.22
1245.38725	7	$(c_{75}+5\mathrm{Ac})^{7+\bullet}$	0.41	10.27
1267.67878	2	$(c_{22}+2\mathrm{Ac})^{2+}$	3.18	4.22
1280.70156	2	$(a_{23}+\mathrm{Ac})^{2+}$	0.62	2.23
1281.04033	3	$y_{34}{}^{3+}$	-0.48	2.76
1389.77616	1	$(c_{11}+3\mathrm{Ac})^{+\bullet}$	5.54	9.21
1405.76034	4	$(c_{49}+3\mathrm{Ac})^{4+\bullet}$	-0.45	5.02
1485.78705	2	$y_{26}{}^{2+}$	-2.94	2.92
1486.78961	2	$(b_{26}+2\mathrm{Ac})^{2+}$	6.15	29.41
1491.83256	1	$(c_{12}+3\mathrm{Ac})^{+}$	5.76	5.57
1498.79693	2	$(z_{26}+\mathrm{Ac})^{2+}$	6.40	5.95

Average mass accuracy: 1.67 ppm (standard deviation: 1.53 ppm)

Table S3. Peak assignments for the $[M+9H+4Ac]^{9+}$ fragment ion scan (*m/z* 972).

m/z ratio	*z*	name	Mass accuracy (ppm)	S/N
386.22728	1	z_4	0.15	6.59
640.04106	3	z_{17}^{3+}	-1.07	45.18
653.39683	2	z_{12}^{2+}	-0.90	7.91
717.91856	2	z_{13}^{2+}	-0.21	13.11
833.12643	3	z_{22}^{2+}	0.00	5.36
847.12943	3	$(z_{22}+Ac)^{3+}$	-0.61	27.82
904.49560	1	$(a_7+2Ac)^{+}$	-0.54	8.32
942.50988	3	z_{25}^{3+}	0.30	12.09
949.52310	1	$(c_7+2Ac)^{+}$	5.84	4.53
956.51197	3	$(z_{25}+Ac)^{3+}$	-1.20	121.33
966.07578	9	$[M+9H+3Ac]^{9+}$	-3.66	5.41
970.74645	9	$[M+9H+4Ac]^{9+}$	-0.74	21117.36
982.19376	3	$(a_{26}+2Ac)^{3+}$	2.70	21.60
993.03649	4	$(a_{34}+4Ac)^{4+}$	2.53	6.65
993.40900	8	$(y_{70}+3Ac)^{8+}$	-2.47	9.25
999.52274	3	$(z_{26}+Ac)^{3+}$	-4.58	8.67
999.85779	3	$(z_{26}+Ac)^{3+\bullet}$	-5.47	6.40
1012.34794	5	$(z_{44}+2Ac)^{5+}$	1.20	22.02
1016.39975	7	$(b_{62}+3Ac)^{7+}$	4.99	26.13
1025.89116	3	$(c_{27}+Ac)^{3+}$	-1.85	8.42
1032.72293	6	$(c_{54}+3Ac)^{6+}$	-0.78	139.66
1035.55314	5	$(z_{45}+2Ac)^{5+\bullet}$	-0.52	74.90
1038.55717	5	$(y_{45}+2Ac)^{5+}$	1.26	19.40
1043.75319	5	$(z_{45}+3Ac)^{5+}$	-0.99	62.85
1043.95473	5	$(z_{45}+3Ac)^{5+\bullet}$	-1.01	55.06
1049.32247	4	$(a_{37}+3Ac)^{4+}$	1.49	7.86
1049.39705	6	$(c_{55}+3Ac)^{6+\bullet}$	0.00	9.56
1055.42476	7	$(z_{65}+2Ac)^{7+}$	1.90	13.64
1055.56802	7	$(z_{65}+2Ac)^{7+\bullet}$	1.22	17.77
1057.40418	6	$(z_{55}+3Ac)^{6+}$	-1.37	5.26
1057.57186	6	$(z_{55}+3Ac)^{6+\bullet}$	-1.64	5.59
1060.07487	6	$(y_{55}+3Ac)^{6+}$	-0.52	37.09
1060.96262	5	$(z_{46}+2Ac)^{5+}$	-1.13	12.15
1061.16370	5	$(z_{46}+2Ac)^{5+\bullet}$	-1.60	33.20
1062.59693	2	$(z_{18}+Ac)^{2+\bullet}$	-2.73	11.72
1073.58427	8	$(z_{75}+4Ac)^{8+}$	1.88	126.31
1074.33736	4	$(c_{38}+2Ac)^{4+}$	0.50	58.41
1080.08361	2	$(b_{18}+3Ac)^{2+}$	-3.18	8.83
1084.71343	8	$(c_{75}+4Ac)^{8+}$	-0.49	369.09
1086.71307	8	$[M+8H+3Ac]^{8+}$	-0.24	2699.43
1089.96389	8	$[M+8H+4Ac-NH_3]^{8+\bullet}$	1.45	1236.87
1092.08725	8	$[M+8H +4Ac]^{8+\bullet}$	-3.09	1425.59
1099.40056	5	$(c_{48}+3Ac)^{5+}$	0.44	4.41
1101.92116	6	$(c_{58}+3Ac)^{6+\bullet}$	0.25	7.41
1102.08848	6	$(c_{58}+3Ac)^{6+}$	-0.35	13.17

1109.84171	4	$(z_{38}$+2Ac$)^{4+}$	-2.46	8.33
1110.09639	4	$(z_{38}$+2Ac$)^{4+\bullet}$	0.00	4.52
1120.80167	5	$(y_{48}$+3Ac$)^{5+}$	-3.41	9.14
1121.76214	6	$(z_{59}$+2Ac$)^{6+}$	-0.70	6.81
1121.92926	6	$(z_{59}$+2Ac$)^{6+\bullet}$	-1.46	9.22
1124.02252	7	$(c_{68}$+4Ac$)^{7+\bullet}$	0.68	8.81
1129.26544	6	$(c_{59}$+3Ac$)^{6+}$	-0.57	125.43
1143.96685	3	$(c_{30}$+2Ac$)^{3+}$	-1.55	13.83
1147.62972	5	$(c_{50}$+3Ac$)^{5+}$	0.97	24.32
1154.47311	7	$(c_{70}$+4Ac$)^{7+}$	-0.11	68.13
1155.61744	3	$(z_{30}$+2Ac$)^{3+}$	0.21	5.49
1157.19134	7	$(z_{71}$+4Ac$)^{7+}$	-3.93	10.34
1157.34244	7	$(z_{71}$+4Ac$)^{7+\bullet}$	2.23	25.45
1159.34475	7	$(z_{72}$+2Ac$)^{7+}$	-0.65	32.20
1159.95283	6	$(z_{61}$+2Ac$)^{6+\bullet}$	2.96	9.62
1162.45145	6	$(y_{61}$+2Ac$)^{6+}$	0.21	5.92
1167.12239	6	$(c_{61}$+3Ac$)^{6+}$	1.55	10.31
1172.65187	3	$(c_{31}$+Ac$)^{3+}$	0.49	41.92
1173.43800	5	$(c_{51}$+3Ac$)^{5+}$	0.75	292.26
1177.65184	1	$(c_{10}$+Ac$)^{+\bullet}$	-0.45	7.40
1181.84200	5	$(c_{51}$+4Ac$)^{5+}$	2.33	78.76
1186.65451	3	$(c_{31}$+2Ac$)^{3+}$	-0.26	6.46
1188.30079	6	$(y_{62}$+3Ac$)^{6+}$	0.40	59.34
1188.46813	6	$(c_{62}$+3Ac$)^{6+}$	3.75	46.88
1192.35797	7	$(z_{73}$+4Ac$)^{7+}$	-0.27	63.87
1192.92936	7	$(c_{72}$+4Ac$)^{7+}$	0.93	248.41
1200.25135	5	$(z_{52}$+3Ac$)^{5+\bullet}$	-0.54	3.73
1206.14849	4	$(c_{42}$+2Ac$)^{4+}$	0.30	43.11
1209.08404	7	$(c_{73}$+4Ac$)^{7+}$	0.76	134.80
1216.98385	6	$(y_{64}$+2Ac$)^{6+}$	-2.73	38.36
1219.66624	2	$(z_{21}$+Ac$)^{2+}$	-0.98	7.85
1226.80735	7	$(z_{75}$+4Ac$)^{7+}$	0.09	129.02
1226.95083	7	$(z_{75}$+4Ac$)^{7+\bullet}$	-0.32	134.80
1233.53198	7	$(c_{75}$+3Ac$)^{7+}$	3.48	15.10
1238.32264	6	$(c_{64}$+4Ac$)^{6+}$	0.77	130.10
1238.65633	6	$(c_{65}$+2Ac$)^{6+\bullet}$	0.64	55.56
1239.52581	7	$[M+7H+3Ac-NH_3]^{7+\bullet}$	0.01	383.73
1241.81231	7	$[M+7H+3Ac]^{7+}$	-1.51	114.23
1270.19679	2	$(z_{22}$+Ac$)^{2+}$	4.34	8.14
1288.17363	2	$(c_{22}$+3Ac$)^{2+\bullet}$	-1.93	6.84
1299.29829	5	$(c_{57}$+3Ac$)^{5+}$	-1.15	12.42
1299.45895	4	$(c_{45}$+2Ac$)^{4+}$	1.30	41.55
1310.71564	3	$(c_{34}$+2Ac$)^{3+}$	4.57	8.04
1363.32267	5	$(c_{59}$+4Ac$)^{5+}$	1.99	108.22
1446.45166	3	$(y_{37}$+2Ac$)^{3+}$	-1.67	17.35
1465.77579	2	$(b_{26}$+Ac$)^{2+}$	0.41	17.95
1465.78745	3	$(z_{38}$+Ac$)^{3+\bullet}$	-1.22	54.80

Average mass accuracy: 1.47 ppm (standard deviation: 1.35 ppm)

Table S4. Peak assignments for the $[M+9H+5Ac]^{9+}$ fragment ion scan (*m/z* 976).

m/z ratio	*z*	name	Mass accuracy (ppm)	S/N
386.22724	1	z_4	0.05	9.17
653.39626	2	z_{12}^{2+}	-1.77	16.54
655.41104	1	z_6	-2.06	4.14
717.91914	2	z_{13}^{2+}	0.59	17.05
816.44173	3	$(a_{22}+Ac)^{3+}$	0.18	6.76
847.12959	3	$(z_{22}+Ac)^{3+}$	-0.42	45.58
855.13990	3	c_{23}^{3+}	0.00	4.25
889.01786	2	$(c_{15}+2Ac)^{2+}$	-0.43	17.09
921.49032	4	$(z_{32}+2Ac)^{4+}$	-0.84	19.60
921.61515	8	$(b_{64}+3Ac)^{8+}$	1.84	20.43
956.51248	3	$(z_{25}+Ac)^{3+}$	-0.67	241.24
961.52262	4	$(c_{33}+3Ac)^{4+}$	-3.57	6.79
970.74580	9	$[M+9H+4Ac]^{9+}$	-1.41	115.98
975.41273	9	$[M+9H+5Ac]^{9+}$	-2.33	39681.83
978.69186	6	$(z_{50}+5Ac)^{6+}$	-3.08	12.13
980.56254	2	$(z_{17}+Ac)^{2+}$	-1.75	25.72
986.72975	5	$(z_{43}+2Ac)^{5+}$	2.04	16.33
988.90911	8	$(a_{70}+Ac)^{8+}$	0.78	5.08
999.52751	3	$(z_{26}+Ac)^{3+}$	0.19	11.51
1000.21025	6	$(z_{52}+3Ac)^{6+}$	0.34	59.59
1001.95141	5	$(c_{44}+Ac)^{5+}$	-0.05	10.49
1003.07347	2	$(c_{17}+2Ac)^{2+}$	-0.28	40.90
1005.62854	1	$z_9^{+\bullet}$	-3.05	6.75
1011.88997	3	c_{27}^{3+}	0.43	9.72
1016.40069	7	$(z_{62}+3Ac)^{7+}$	2.02	14.66
1018.75589	5	$(c_{44}+3Ac)^{5+}$	0.20	12.16
1032.72686	6	$(c_{54}+3Ac)^{6+}$	3.02	4.33
1035.35289	5	$(z_{45}+2Ac)^{5+}$	0.75	23.47
1035.55345	5	$(z_{45}+2Ac)^{5+\bullet}$	-0.22	10.90
1039.72718	6	$(c_{54}+4Ac)^{6+}$	1.61	221.50
1043.75368	5	$(z_{45}+3Ac)^{5+}$	-0.52	165.16
1049.32442	4	$(z_{36}+2Ac)^{4+\bullet}$	-0.56	29.57
1056.56980	6	$(c_{55}+4Ac)^{6+}$	2.85	36.11
1060.07536	6	$(y_{55}+3Ac)^{6+}$	-0.05	103.98
1061.28208	7	$(y_{64}+5Ac)^{7+}$	1.68	10.26
1061.42497	7	$(z_{65}+3Ac)^{7+}$	0.67	8.67
1061.56824	7	$(z_{65}+3Ac)^{7+\bullet}$	0.00	7.65
1062.09582	2	$(z_{18}+Ac)^{2+}$	-0.10	17.26
1063.57600	3	$(c_{28}+2Ac)^{3+}$	0.36	34.64
1069.36680	5	$(z_{46}+3Ac)^{5+}$	0.81	14.71
1069.56677	5	$(z_{46}+3Ac)^{5+\bullet}$	-0.69	14.21
1079.74874	6	$(y_{57}+Ac)^{6+}$	0.32	6.97
1084.84309	4	$(c_{38}+3Ac)^{4+}$	3.34	73.90
1085.09372	4	$(y_{37}+2Ac)^{4+}$	1.24	31.90

1085.90972	3	$(b_{28}$+4Ac$)^{3+}$	2.37	9.50
1089.83883	8	$(c_{75}$+5Ac$)^{8+\bullet}$	-0.43	112.87
1089.92008	6	$(c_{57}$+4Ac$)^{6+}$	0.56	101.00
1089.96416	8	$(c_{75}$+5Ac$)^{8+}$	-1.03	88.20
1091.96477	8	[M+8H+4Ac]$^{8+}$	0.11	28.34
1095.21302	8	[M+8H+5Ac-NH_3]$^{8+\bullet}$	-0.56	1220.42
1099.59645	5	$c_{49}{}^{5+\bullet}$	-6.77	5.20
1106.27496	3	$(c_{29}$+2Ac$)^{3+}$	0.93	38.65
1109.08987	6	$(c_{58}$+4Ac$)^{6+}$	-0.68	14.28
1109.84792	4	$(z_{38}$+2Ac$)^{4+}$	3.15	22.74
1117.80344	5	$(z_{48}$+3Ac$)^{5+\bullet}$	0.12	40.81
1119.93776	3	$(c_{29}$+3Ac$)^{3+\bullet}$	-3.35	15.78
1120.11010	2	$(z_{19}$+Ac$)^{2+\bullet}$	-2.86	5.32
1120.80775	5	$(y_{48}$+3Ac$)^{5+}$	2.02	14.35
1128.76274	6	$(z_{59}$+3Ac$)^{6+}$	-1.72	107.86
1130.02592	7	$(c_{68}$+5Ac$)^{7+\bullet}$	2.34	12.09
1131.80821	5	$(z_{49}$+3Ac$)^{5+}$	-0.85	66.75
1136.26688	6	$(c_{59}$+4Ac$)^{6+}$	-0.85	118.58
1139.02819	5	$(c_{50}$+2Ac$)^{5+\bullet}$	2.86	7.14
1155.82893	5	$(c_{50}$+4Ac$)^{5+\bullet}$	-0.20	17.14
1156.03121	5	$(c_{50}$+4Ac$)^{5+}$	0.42	52.94
1159.64402	2	$(c_{20}$+2Ac$)^{2+}$	5.68	6.40
1160.33082	7	$(c_{70}$+5Ac$)^{7+\bullet}$	0.05	45.08
1163.34394	7	$(z_{71}$+5Ac$)^{7+\bullet}$	2.20	21.75
1166.95212	6	$(z_{61}$+3Ac$)^{6+\bullet}$	0.83	84.91
1171.34852	7	$(z_{72}$+4Ac$)^{7+}$	0.00	8.82
1171.49037	7	$(z_{72}$+4Ac$)^{7+\bullet}$	-1.81	6.31
1174.22405	5	$(z_{50}$+5Ac$)^{5+}$	-7.10	5.36
1177.62656	4	$(c_{41}$+3Ac$)^{4+}$	0.91	11.62
1181.83993	5	$(c_{51}$+4Ac$)^{5+}$	0.58	486.59
1186.65754	3	$(c_{31}$+2Ac$)^{3+}$	2.30	105.25
1188.46482	6	$(c_{62}$+3Ac$)^{6+}$	0.96	14.09
1190.44563	5	$(a_{53}$+2Ac$)^{5+}$	4.39	5.73
1195.39588	4	$(c_{42}$+Ac$)^{4+\bullet}$	1.97	8.39
1198.93284	7	$(c_{72}$+5Ac$)^{7+}$	2.56	236.73
1200.66318	3	$(c_{31}$+3Ac$)^{3+}$	4.04	31.41
1215.08459	7	$(c_{73}$+5Ac$)^{7+}$	-0.03	192.72
1216.24963	5	$(c_{53}$+4Ac$)^{5+}$	0.58	31.46
1216.65228	4	$(c_{42}$+3Ac$)^{4+}$	1.25	105.36
1220.67666	1	$(c_{10}$+2Ac$)^{+}$	4.84	5.35
1224.15190	6	$(z_{65}$+Ac$)^{6+}$	-4.09	43.77
1228.31998	3	$(z_{32}$+2Ac$)^{3+}$	0.77	23.49
1232.80992	7	$(z_{75}$+5Ac$)^{7+}$	0.95	185.84
1238.99920	3	$(c_{32}$+3Ac$)^{3+}$	-1.17	10.42
1241.81624	7	[M+7H+3Ac]$^{7+}$	1.65	7.17
1245.32479	6	$(c_{64}$+5Ac$)^{6+}$	1.07	185.94
1245.52630	7	$(c_{75}$+5Ac$)^{7+}$	-3.54	478.60

1247.47003	5	$(c_{54}$+4Ac$)^{5+}$	0.71	169.05
1270.19336	2	$(z_{22}$+Ac$)^{2+}$	1.64	37.71
1270.69966	2	$(z_{22}$+Ac$)^{2+\bullet}$	3.52	22.11
1273.19380	4	$(c_{44}$+3Ac$)^{4+}$	0.79	32.67
1278.20064	2	$(y_{22}$+Ac$)^{2+}$	0.00	3.67
1295.45930	4	$(a_{46}$+Ac$)^{4+}$	0.60	4.84
1307.50034	5	$(c_{57}$+4Ac$)^{5+\bullet}$	0.00	31.86
1309.96157	4	$(c_{45}$+3Ac$)^{4+}$	1.27	67.54
1348.77037	1	$(c_{11}$+2Ac$)^{+}$	3.45	3.40
1371.72412	5	$(c_{59}$+5Ac$)^{5+}$	1.50	39.13
1388.23846	2	$(c_{24}$+2Ac$)^{2+\bullet}$	3.10	8.26
1388.73975	2	$(c_{24}$+2Ac$)^{2+}$	1.20	21.14
1410.25421	6	$(c_{73}$+4Ac$)^{6+\bullet}$	-4.90	5.82
1452.78430	6	$(c_{75}$+5Ac$)^{6+\bullet}$	0.68	14.44
1452.94766	6	$[M+6H+4Ac-NH_3]^{6+\bullet}$	0.24	14.62
1489.56053	4	$(z_{52}$+2Ac$)^{4+\bullet}$	0.00	4.13

Average mass accuracy: 1.52 ppm (standard deviation: 1.49 ppm)

Table S5. Peak assignments for the $[M+9H+6Ac]^{9+}$ fragment ion scan (*m/z* 981).

m/z ratio	*z*	name	Mass accuracy (ppm)	S/N
432.22714	1	$(c_3+Ac)^+$	-0.87	2.92
868.56899	1	$z_8^{+\bullet}$	-4.27	7.74
902.28984	5	$(y_{39}+Ac)^{5+}$	0.73	7.44
904.49205	1	$(a_7+2Ac)^+$	-4.46	6.22
912.49522	7	$(z_{55}+4Ac)^{7+}$	2.34	6.42
924.50050	6	$(z_{48}+2Ac)^{6+}$	-0.44	5.30
937.67767	6	$(c_{49}+3Ac)^{6+}$	-0.05	8.33
970.74247	9	$[M+9H+4Ac]^{9+}$	-4.84	30.14
975.41147	9	$[M+9H+5Ac]^{9+}$	-3.62	98.60
978.07852	9	$[M+9H+6Ac-H_2O]^{9+}$	-3.22	8.75
1043.75422	5	$(z_{45}+3Ac)^{5+}$	0.00	10.35
1046.72728	6	$(c_{54}+5Ac)^{6+}$	0.02	29.99
1060.07423	6	$(y_{55}+3Ac)^{6+}$	-1.12	8.09
1095.33776	4	$(c_{38}+4Ac)^{4+}$	-3.97	28.59
1100.46532	8	$[M+8H+6Ac-NH_3]^{8+\bullet}$	0.34	406.48
1107.59721	5	$(c_{48}+4Ac)^{5+\bullet}$	-3.09	8.00
1116.58449	7	$(c_{67}+6Ac)^{7+}$	-3.95	6.80
1119.84473	4	$(b_{39}+4Ac)^{4+}$	2.26	6.73
1132.00646	5	$(z_{49}+3Ac)^{5+\bullet}$	-3.77	8.71
1138.63267	2	$(c_{20}+Ac)^{2+}$	0.45	4.75
1163.12541	2	$(z_{20}+Ac)^{2+}$	0.00	9.79
1190.24226	5	$(c_{51}+5Ac)^{5+}$	0.76	118.91
1194.98439	3	$(b_{31}+3Ac)^{3+}$	1.31	8.24
1198.36216	7	$(z_{73}+5Ac)^{7+}$	1.97	123.03
1200.32172	3	$(c_{31}+3Ac)^{3+\bullet}$	-0.56	8.19
1200.65959	3	$(c_{31}+3Ac)^{3+}$	1.05	7.21
1204.78617	7	$(c_{72}+6Ac)^{7+\bullet}$	-0.94	9.81
1204.93107	7	$(c_{72}+6Ac)^{7+}$	-0.17	33.04
1214.65231	7	$(z_{74}+5Ac)^{7+\bullet}$	-5.22	4.07
1216.64700	2	$(c_{21}+2Ac)^{2+\bullet}$	0.00	11.25
1220.94217	7	$(c_{73}+6Ac)^{7+\bullet}$	0.00	27.79
1249.69177	2	$z_{22}^{2+\bullet}$	1.48	4.90
1252.66111	3	$(c_{32}+4Ac)^{3+\bullet}$	-5.69	33.49
1252.66123	6	$(c_{65}+4Ac)^{6+\bullet}$	1.73	7.38
1381.23688	2	a_{25}^{2+}	-0.99	6.19
1478.79075	3	$(b_{39}+3Ac)^{3+}$	4.80	8.68

Average mass accuracy: 1.96 ppm (standard deviation: 1.77 ppm)

Table S6. Peak assignments for the $[M+8H+4Ac]^{8+}$ fragment ion scan (*m/z* 1093).

m/z ratio	*z*	name	Mass accuracy (ppm)	S/N
661.40673	2	y_{12}^{2+}	-0.08	2.62
833.12643	3	z_{22}^{3+}	0.00	5.47
867.56268	1	z_8^{+}	-2.53	8.25
930.01813	4	c_{33}^{4+}	0.00	6.70
1039.89867	3	$(c_{27}+2Ac)^{3+}$	2.01	5.64
1089.83395	8	$[M+8H+4Ac-NH_3]^{8+}$	-2.18	5.83
1091.96343	8	$[M+8H+4Ac]^{8+}$	-1.11	23484.30
1118.71987	1	$z_{10}^{+\bullet}$	3.75	3.54
1124.09955	4	$(c_{39}+4Ac)^{4+}$	0.63	5.30
1185.62636	6	$(b_{62}+3Ac)^{6+}$	0.37	3.91
1226.80792	7	$(z_{75}+4Ac)^{7+}$	0.55	147.37
1230.66760	5	$(c_{54}+2Ac)^{5+}$	2.18	9.00
1233.38868	7	$(c_{75}+3Ac)^{7+\bullet}$	4.02	5.88
1239.38624	7	$(c_{75}+4Ac)^{7+\bullet}$	0.81	186.50
1239.52947	7	$(c_{75}+4Ac)^{7+}$	0.22	282.19
1241.81410	7	$[M+7H+3Ac]^{7+}$	-0.07	974.49
1247.96036	7	$[M+7H+4Ac]^{7+\bullet}$	0.55	3696.86
1250.27179	5	$(a_{55}+3Ac)^{5+}$	-0.39	3.14
1259.69548	2	a_{23}^{2+}	0.00	4.47
1262.67087	6	$(c_{66}+3Ac)^{6+}$	3.42	6.60
1265.18209	4	$(z_{44}+2Ac)^{4+}$	0.40	8.72
1270.19027	2	$(z_{22}+Ac)^{2+}$	-0.80	6.22
1290.89522	5	$(c_{57}+2Ac)^{5+}$	-1.90	14.69
1299.30290	5	$(c_{57}+3Ac)^{5+}$	2.39	15.94
1304.44705	4	$(z_{45}+3Ac)^{5+}$	4.67	19.96
1311.35932	6	$(c_{68}+4Ac)^{6+}$	0.39	53.23
1346.54959	6	$(c_{70}+4Ac)^{6+\bullet}$	0.00	7.98
1347.72595	4	$(y_{47}+Ac)^{4+}$	-0.05	3.44
1354.92034	5	$(c_{59}+3Ac)^{5+}$	1.84	44.55
1363.32140	5	$(c_{59}+4Ac)^{5+}$	1.06	28.70
1390.91795	6	$(z_{73}+4Ac)^{6+}$	0.83	32.80
1391.58538	6	$(c_{72}+4Ac)^{6+}$	2.61	99.71
1396.25564	6	$(c_{73}+2Ac)^{6+\bullet}$	-1.41	4.75
1409.76991	6	$(z_{74}+4Ac)^{6+}$	4.09	44.96
1410.43057	6	$(c_{73}+4Ac)^{6+}$	1.04	91.37
1425.96305	5	$(z_{63}+Ac)^{5+}$	1.80	17.87
1431.10645	6	$(z_{75}+4Ac)^{6+}$	-0.55	11.44
1431.27492	6	$(z_{75}+4Ac)^{6+\bullet}$	-0.20	20.02
1441.12297	3	$(z_{37}+2Ac)^{3+}$	5.88	4.45
1441.27733	4	$(b_{51}+Ac)^{4+}$	-3.71	6.80
1441.45599	3	$(z_{37}+2Ac)^{3+\bullet}$	3.85	6.72
1445.95205	6	$(c_{75}+4Ac)^{6+}$	1.75	158.48
1452.78413	6	$[M+6H+4Ac-H_2O]^{6+\bullet}$	0.56	25.13
1452.95046	6	$[M+6H+4Ac-H_2O]^{6+\bullet\bullet}$	-0.57	55.75
1453.12155	6	$[M+6H+4Ac-NH_3]^{6+\bullet\bullet}$	4.31	38.09

1456.46004	3	$(c_{39}+Ac)^{3+}$	5.43	40.07
1466.54839	4	$(c_{51}+3Ac)^{4+}$	2.59	21.41
1466.79607	4	a_{53}^{4+}	1.81	24.71
1538.33517	2	$(c_{27}+Ac)^{2+}$	-0.50	7.88
1734.74071	5	$(c_{75}+4Ac)^{5+\bullet}$	2.48	4.63
1734.94122	5	$[M+5H+3Ac-H_2O]^{5+\bullet}$	1.87	9.48

Average mass accuracy: 1.69 ppm (standard deviation: 1.54 ppm)

Table S7. Peak assignments for the [M+8H+5Ac]$^{8+}$ fragment ion scan (*m/z* 1098).

m/z ratio	*z*	name	Mass accuracy (ppm)	S/N
386.22807	1	z_4	2.19	39.38
390.21790	1	c_3	2.43	15.32
402.24625	1	y_4	0.77	2.08
537.28530	1	c_4	-0.12	7.94
609.87989	2	z_{11}	-2.48	9.94
636.35322	1	c_5	-0.89	14.05
653.39775	2	z_{12}	0.51	23.51
655.41149	1	z_6	-1.39	4.65
717.91913	2	z_{13}	0.58	7.94
806.45740	1	$(c_6\text{+Ac})^{+}$	-2.37	10.08
833.12400	3	z_{22}	-2.91	9.46
838.46600	3	y_{22}	0.00	10.33
847.12995	3	$(z_{22}\text{+Ac})^{3+}$	0.00	9.98
848.47073	1	$(c_6\text{+2Ac})^{+}$	1.01	5.92
867.00085	2	$(z_{15}\text{+Ac})^{2+}$	0.09	21.46
867.56305	1	z_8	-2.10	6.08
899.16378	3	$(z_{23}\text{+Ac})^{3+}$	0.14	47.85
907.50746	1	$(c_7\text{+Ac})^{+}$	0.53	9.75
910.99386	4	$(z_{32}\text{+Ac})^{4+}$	5.93	5.99
914.74061	4	$(b_{32}\text{+2Ac})^{4+}$	-2.65	4.40
921.49107	4	$(z_{32}\text{+2Ac})^{4+}$	-0.03	22.95
942.50937	3	z_{25}^{3+}	-0.24	21.56
956.51275	3	$(z_{25}\text{+Ac})^{3+}$	-0.38	376.68
965.11775	5	$(c_{42}\text{+2Ac})^{5+}$	-2.29	4.08
978.03217	4	$(z_{34}\text{+2Ac})^{4+}$	-0.98	24.63
980.56156	2	$(z_{17}\text{+Ac})^{2+}$	-2.75	70.32
985.52297	3	z_{26}^{3+}	-0.84	16.41
999.52710	3	$(z_{26}\text{+Ac})^{3+}$	-0.21	126.11
1012.34763	5	$(z_{44}\text{+2Ac})^{5+}$	0.89	34.76
1017.05960	4	$(z_{35}\text{+2Ac})^{4+}$	1.17	51.31
1018.39924	7	$(a_{62}\text{+4Ac})^{7+}$	2.28	4.70
1022.36387	5	$(a_{45}\text{+Ac})^{5+}$	2.95	4.68
1035.35388	5	$(z_{45}\text{+2Ac})^{5+}$	1.70	48.66
1035.55518	5	$(c_{44}\text{+5Ac})^{5+}$	-4.57	62.95
1044.15574	5	z_{46}^{5+}	-3.69	15.20
1049.07004	4	$(z_{36}\text{+2Ac})^{4+}$	-2.87	6.99
1049.32045	4	$(a_{37}\text{+3Ac})^{4+}$	-0.43	7.53
1057.89621	3	$(b_{28}\text{+2Ac})^{3+}$	-3.67	5.81
1060.96446	5	$(z_{46}\text{+2Ac})^{5+}$	0.60	94.00
1062.09688	2	$(z_{18}\text{+Ac})^{2+}$	0.90	98.37
1067.90745	3	$(c_{27}\text{+4Ac})^{3+}$	3.58	5.05
1069.36423	5	$(z_{46}\text{+3Ac})^{5+}$	-1.59	6.86
1070.10389	2	$(y_{18}\text{+Ac})^{2+}$	-1.31	12.57
1073.17891	5	$(a_{47}\text{+4Ac})^{5+}$	0.00	6.67
1079.90722	3	$(z_{28}\text{+Ac})^{3+}$	-0.91	81.34

1080.58145	6	$(a_{58}+\mathrm{Ac})^{6+}$	-0.29	16.69
1081.08866	4	$(z_{37}+2\mathrm{Ac})^{4+}$	0.90	126.09
1083.58130	5	$(z_{47}+2\mathrm{Ac})^{5+}$	0.61	29.86
1085.24675	3	$(y_{28}+\mathrm{Ac})^{3+}$	-0.94	141.68
1086.71250	8	$[\mathrm{M}+8\mathrm{H}+3\mathrm{Ac}]^{8+}$	-0.76	367.67
1091.96286	8	$[\mathrm{M}+8\mathrm{H}+4\mathrm{Ac}]^{8+}$	-1.64	3937.76
1097.21535	8	$[\mathrm{M}+8\mathrm{H}+5\mathrm{Ac}]^{8+}$	-0.57	136864.08
1105.84675	4	$(c_{38}+5\mathrm{Ac})^{4+}$	1.82	95.24
1109.84533	4	$(z_{38}+2\mathrm{Ac})^{4+}$	0.81	6.14
1117.60039	5	$(z_{48}+3\mathrm{Ac})^{5+}$	-1.22	55.21
1120.11300	2	$(z_{19}+\mathrm{Ac})^{+\bullet}$	-0.28	48.16
1120.80684	5	$(y_{48}+3\mathrm{Ac})^{5+}$	1.21	97.16
1123.34454	4	$(a_{39}+5\mathrm{Ac})^{4+}$	-1.40	7.58
1127.62176	2	$(y_{19}+\mathrm{Ac})^{2+}$	2.66	71.73
1130.11888	2	$(b_{20}+\mathrm{Ac})^{2+}$	0.00	8.48
1131.80986	5	$(z_{49}+3\mathrm{Ac})^{5+}$	0.61	211.54
1133.72727	1	y_{10}	0.62	5.61
1135.63628	1	$c_{10}^{\bullet}$	-4.87	3.26
1136.61413	3	$(z_{29}+2\mathrm{Ac})^{3+}$	3.60	68.56
1138.12824	2	$(c_{20}+\mathrm{Ac})^{2+\bullet}$	0.00	6.40
1143.96907	3	$(c_{30}+2\mathrm{Ac})^{3+}$	0.39	6.83
1145.27689	6	$(z_{60}+3\mathrm{Ac})^{6+}$	0.70	100.62
1155.62147	3	$(z_{30}+2\mathrm{Ac})^{3+}$	3.70	3.88
1155.95572	3	$(z_{30}+2\mathrm{Ac})^{3+\bullet}$	2.24	9.06
1162.37514	4	$(y_{40}+2\mathrm{Ac})^{4+}$	-0.30	22.81
1167.12010	6	$(c_{61}+3\mathrm{Ac})^{6+}$	-0.41	14.47
1179.63638	3	$(z_{31}+2\mathrm{Ac})^{3+\bullet}$	3.56	8.54
1185.63271	6	$(z_{62}+3\mathrm{Ac})^{6+}$	1.84	45.45
1185.80130	6	$(z_{62}+3\mathrm{Ac})^{6+\bullet}$	2.36	72.74
1186.64122	4	$(z_{41}+2\mathrm{Ac})^{4+}$	-0.51	77.64
1186.65774	3	$(c_{31}+2\mathrm{Ac})^{3+}$	2.46	145.87
1186.89338	4	$(z_{41}+2\mathrm{Ac})^{4+\bullet}$	-0.34	54.58
1201.14859	4	$(z_{42}+2\mathrm{Ac})^{4+\bullet}$	-0.47	125.89
1202.47000	6	$(c_{62}+5\mathrm{Ac})^{6+}$	2.33	7.26
1204.90645	4	$(y_{42}+2\mathrm{Ac})^{4+}$	3.79	63.77
1216.65061	4	$(c_{42}+3\mathrm{Ac})^{4+}$	-0.13	34.61
1219.66962	2	$(z_{21}+\mathrm{Ac})^{2+}$	1.78	52.19
1220.17046	2	$(z_{21}+\mathrm{Ac})^{2+\bullet}$	-0.73	29.01
1224.66429	3	$(c_{32}+2\mathrm{Ac})^{3+\bullet}$	2.53	55.82
1226.80905	7	$(z_{75}+4\mathrm{Ac})^{7+}$	1.47	26.33
1226.95230	7	$(z_{75}+4\mathrm{Ac})^{7+\bullet}$	0.88	44.17
1228.32087	3	$(z_{32}+2\mathrm{Ac})^{3+}$	1.50	24.43
1228.65513	3	$(z_{32}+2\mathrm{Ac})^{3+\bullet}$	0.12	34.02
1232.81170	7	$(z_{75}+5\mathrm{Ac})^{7+}$	2.40	212.25
1232.95409	7	$(z_{75}+5\mathrm{Ac})^{7+\bullet}$	1.10	196.03
1233.09643	7	$(a_{75}+4\mathrm{Ac})^{7+}$	-0.92	71.61
1233.41280	4	$(z_{43}+2\mathrm{Ac})^{4+\bullet}$	2.43	114.45

1237.43028	4	b_{44}^{4+}	1.65	7.39
1238.66310	3	$(c_{32}+3Ac)^{3+\bullet}$	-1.30	25.27
1238.86802	5	$(c_{54}+3Ac)^{5+\bullet}$	2.06	87.23
1239.10251	7	$(a_{75}+5Ac)^{7+}$	2.78	63.97
1245.52973	7	$(c_{75}+5Ac)^{7+}$	-0.79	1775.20
1247.81624	7	$[M+7H+4Ac]^{7+}$	0.43	4165.37
1251.53159	7	$[M+7H+5Ac-NH3]^{7+\bullet}$	2.21	1211.02
1253.81888	7	$[M+7H+5Ac]^{7+}$	1.33	3413.72
1253.96216	7	$[M+7H+5Ac]^{7+\bullet}$	0.78	4702.20
1266.01963	3	$(z_{33}+2Ac)^{3+}$	4.66	17.89
1267.68183	5	$(c_{55}+4Ac)^{5+}$	2.48	212.75
1270.19123	2	$(z_{22}+Ac)^{2+}$	-0.04	231.45
1270.69214	2	$(z_{22}+Ac)^{2+\bullet}$	-2.40	85.62
1273.19208	4	$(c_{44}+3Ac)^{4+}$	-0.56	51.45
1275.93827	4	$(z_{44}+3Ac)^{4+\bullet}$	1.64	89.70
1278.20355	2	$(y_{22}+Ac)^{2+}$	2.27	79.87
1281.70177	3	$(c_{33}+3Ac)^{3+}$	2.18	78.89
1288.17612	2	$(c_{22}+3Ac)^{2+\bullet}$	0.00	5.79
1294.19756	4	$(c_{44}+5Ac)^{4+}$	-0.40	5.56
1298.69761	5	$(c_{56}+5Ac)^{5+}$	0.00	3.09
1304.04524	3	$(z_{34}+2Ac)^{3+\bullet}$	0.68	42.57
1304.69253	4	$(z_{45}+3Ac)^{4+\bullet}$	-0.30	211.17
1306.79081	1	$z_{12}^{\bullet}$	-3.50	4.67
1307.70456	5	$(c_{57}+4Ac)^{5+}$	2.03	178.53
1309.29389	5	$(z_{57}+3Ac)^{5+\bullet}$	-3.64	6.45
1311.87305	6	$(z_{69}+4Ac)6+^{\bullet}$	1.16	21.24
1318.36278	6	$(c_{68}+5Ac)^{6+}$	1.68	177.57
1321.54565	6	$(z_{70}+3Ac)^{6+}$	1.95	47.54
1321.71277	6	$(z_{70}+3Ac)^{6+\bullet}$	1.31	94.92
1322.10736	5	$(c_{58}+3Ac)^{5+\bullet}$	2.84	137.15
1324.71311	3	$(c_{34}+3Ac)^{3+}$	-0.04	115.73
1328.54498	6	$(z_{70}+4Ac)^{6+}$	0.11	25.88
1328.71211	6	$(z_{70}+4Ac)^{6+\bullet}$	-0.52	39.24
1330.71235	5	$(c_{58}+4Ac)^{5+}$	3.80	245.66
1336.70720	4	$(z_{46}+3Ac)^{4+\bullet}$	-0.27	43.03
1341.97820	4	$(c_{47}+3Ac)^{4+}$	2.72	63.64
1343.72194	3	$(c_{35}+3Ac)^{3+}$	1.20	103.24
1348.77176	1	$(c_{11}+2Ac)^{+}$	4.48	23.01
1353.71994	6	$(c_{70}+5Ac)^{6+}$	0.46	88.27
1354.51948	5	$(z_{59}+3Ac)^{5+\bullet}$	1.29	22.95
1354.72013	5	$(c_{59}+3Ac)^{5+\bullet}$	2.84	33.16
1357.06399	6	$(z_{71}+5Ac)^{6+\bullet}$	0.19	81.51
1359.56794	6	$(z_{72}+3Ac)^{6+\bullet}$	-2.70	42.86
1363.32511	5	$(c_{59}+4Ac)^{5+}$	3.78	312.23
1364.97939	4	$(z_{47}+3Ac)^{4+\bullet}$	0.59	34.18
1373.41022	6	$(z_{72}+5Ac)^{6+}$	2.23	43.30

1373.57735	6	$(z_{72}$+5Ac$)^{6+•}$	1.62	42.43
1373.74933	4	$(c_{48}$+3Ac$)^{4+•}$	2.19	267.88
1384.50455	4	$(c_{48}$+4Ac$)^{4+}$	2.61	71.53
1384.75521	4	$(c_{49}$+Ac$)^{4+•}$	-0.35	75.78
1385.93095	5	$(c_{60}$+4Ac$)^{5+•}$	2.87	7.04
1391.58493	6	$(c_{72}$+4Ac$)^{6+}$	2.29	8.34
1397.92102	6	$(z_{73}$+5Ac$)^{6+}$	1.76	76.21
1398.58812	6	$(c_{72}$+5Ac$)^{6+}$	3.29	466.31
1400.13860	5	$(z_{61}$+3Ac$)^{5+•}$	-0.95	8.17
1408.74621	5	$(c_{61}$+4Ac$)^{5+}$	0.61	70.77
1409.76860	6	$(z_{74}$+4Ac$)^{6+}$	3.16	8.93
1409.93573	6	$(z_{74}$+4Ac$)^{6+•}$	2.56	69.08
1414.76634	4	$(z_{49}$+3Ac)4+$^{•}$	3.35	83.83
1417.43469	6	$(c_{73}$+5Ac$)^{6+}$	2.70	397.60
1431.27801	6	$(z_{75}$+4Ac$)^{6+•}$	1.96	12.58
1434.56707	5	$(z_{63}$+2Ac$)^{5+•}$	2.02	29.57
1434.77029	5	$(c_{63}$+Ac$)^{5+}$	0.40	17.67
1434.77175	2	$(z_{25}$+Ac$)^{2+•}$	1.25	51.23
1438.27927	6	$(z_{75}$+5Ac$)^{6+•}$	1.60	244.02
1441.45372	3	$(z_{37}$+2Ac$)^{3+•}$	2.27	28.20
1444.79005	4	$(c_{50}$+4Ac$)^{4+}$	2.40	224.08
1445.78884	6	$(c_{75}$+4Ac$)^{6+•}$	5.04	160.22
1445.95507	6	$(c_{75}$+4Ac$)^{6+}$	3.84	57.03
1446.12318	3	$(c_{38}$+3Ac$)^{3+}$	4.38	38.23
1446.45723	3	$(y_{37}$+2Ac$)^{3+}$	2.18	111.41
1452.95178	6	$(c_{75}$+5Ac$)^{6+}$	0.34	243.37
1453.12021	6	[M+6H+4Ac-NH3]$^{6+••}$	3.39	449.64
1460.12123	6	[M+6H +5Ac-NH3]$^{6+••}$	2.86	823.14
1462.78236	6	[M+6H +5Ac]$^{6+•}$	-3.06	129.35
1469.44155	3	$(a_{39}$+3Ac$)^{3+}$	-7.12	347.26
1477.05244	4	$(c_{51}$+4Ac$)^{4+}$	3.53	791.68
1484.45952	3	$(c_{39}$+3Ac$)^{3+}$	0.23	144.37
1494.19406	5	$(z_{65}$+4Ac$)^{5+•}$	-0.45	174.33
1505.80940	4	$(c_{52}$+4Ac$)^{4+}$	3.61	64.74
1511.39236	5	$(c_{65}$+5Ac$)^{5+•}$	0.54	19.72
1520.06504	4	$(c_{53}$+4Ac$)^{4+}$	3.75	42.08
1548.58620	4	$(c_{54}$+3Ac$)^{4+}$	2.73	30.67
1559.34416	2	$(c_{27}$+2Ac$)^{2+}$	1.88	208.12
1569.83430	3	$(c_{41}$+3Ac$)^{3+}$	1.75	31.87
1573.35829	2	$(c_{28}$+Ac$)^{2+•}$	4.89	21.81
1574.04893	5	$(z_{69}$+4Ac$)^{5+•}$	2.89	9.28
1580.35351	2	$(c_{27}$+3Ac$)^{2+}$	4.43	10.10
1585.85652	5	$(z_{70}$+3Ac$)^{5+•}$	2.98	25.76
1594.87016	2	$(c_{28}$+2Ac$)^{2+}$	6.50	62.87
1601.87061	4	$(c_{56}$+3Ac$)^{4+•}$	4.78	4.71

1606.36499	2	y_{28}^{2+}	1.40	7.03
1621.87423	3	$(c_{42}$+3Ac$)^{3+}$	5.53	99.12
1623.87813	4	$(c_{57}$+3Ac$)^{4+}$	3.21	21.24
1624.06701	5	$(c_{70}$+5Ac$)^{5+\bullet}$	4.22	8.51
1634.38214	4	$(c_{57}$+4Ac$)^{4+}$	4.03	58.70
1644.21064	3	$(z_{43}$+2Ac$)^{3+\bullet}$	0.00	3.84
1658.91593	2	$(c_{29}$+2Ac$)^{2+}$	5.22	26.80
1663.95678	1	$(c_{14}$+2Ac$)^{+}$	7.00	17.34
1669.10976	5	$(z_{73}$+4Ac$)^{5+\bullet}$	5.69	5.13
1669.50806	5	$(c_{72}$+4Ac$)^{5+\bullet}$	7.78	18.86
1678.41271	2	$(a_{29}$+4Ac$)^{2+}$	3.35	7.45
1679.91317	2	$(c_{29}$+3Ac$)^{2+}$	0.37	20.52
1697.26012	3	$(c_{44}$+3Ac$)^{3+}$	3.23	92.96
1703.39380	4	$(z_{59}$+4Ac$)^{4+\bullet}$	-2.45	9.48
1703.65461	4	$(c_{59}$+4Ac$)^{4+\bullet}$	4.96	63.36
1711.26438	3	$(c_{44}$+4Ac$)^{3+}$	3.64	4.79
1714.15796	4	$(c_{59}$+5Ac$)^{4+\bullet}$	5.34	96.05
1715.45183	2	$(c_{30}$+2Ac$)^{2+}$	1.48	23.24
1735.96049	2	$(a_{31}$+Ac$)^{2+}$	-1.20	27.25
1736.96495	2	$c_{31}^{2+\bullet}$	0.49	32.28
1739.26656	3	$(z_{45}$+3Ac$)^{3+\bullet}$	6.76	11.13
1739.60082	3	z_{46}^{3+}	3.64	32.12
1743.54118	5	$[M+5H+4Ac-NH_3]^{5+\bullet\bullet}$	2.46	55.71
1743.74170	5	$[M+5H+4Ac-NH_3]^{5+\bullet\bullet\bullet}$	1.86	156.74
1779.49140	2	$(c_{31}$+2Ac$)^{2+}$	7.20	50.16
1789.30200	3	$c_{48}^{3+\bullet}$	-7.51	6.84
1800.49816	2	$(c_{31}$+3Ac$)^{2+}$	7.94	48.73
1835.98839	4	$(z_{65}$+Ac$)^{4+\bullet}$	2.57	23.95
1856.99280	4	$(z_{65}$+3Ac$)^{4+\bullet}$	2.06	8.34
1906.07400	1	$(c_{16}$+2Ac$)^{+}$	1.16	23.22
1932.01789	4	$(c_{67}$+4Ac$)^{4+\bullet}$	0.00	5.19

Average mass accuracy: 2.08 ppm (standard deviation: 1.73 ppm)

Table S8. Peak assignments for the $[M+8H+6Ac]^{8+}$ fragment ion scan (*m/z* 1103).

m/z ratio	*z*	name	Mass accuracy (ppm)	S/N
867.56317	1	z_8^+	-1.96	7.43
879.23584	4	$(c_{30}+4Ac)^{4+}$	2.58	7.53
1063.25964	3	a_{29}^{3+}	-0.10	5.40
1091.96316	8	$[M+8H+4Ac]^{8+}$	-1.37	27.94
1093.87098	7	$(z_{67}+4Ac)^{7+}$	0.00	4.90
1097.21667	8	$[M+8H+5Ac]^{8+}$	0.64	231.02
1099.33611	4	$(z_{38}+Ac)^{4+}$	-5.16	50.46
1100.21294	8	$[M+8H+6Ac-H_2O]^{8+}$	-2.75	4.27
1102.46678	8	$[M+8H+6Ac]^{8+}$	-0.47	16167.74
1132.81297	5	$(a_{49}+5Ac)^{5+}$	1.09	9.10
1132.95067	3	y_{30}^{3+}	0.84	5.98
1223.66088	4	$(c_{43}+Ac)^{4+\bullet}$	-2.99	4.57
1251.53325	7	$(c_{75}+6Ac)^{7+}$	0.82	11.63
1255.87536	5	$(c_{54}+5Ac)^{5+}$	3.27	83.39
1257.53348	7	$[M+7H+6Ac-NH_3]^{7+\bullet}$	2.51	664.00
1260.28553	5	$(z_{55}+2Ac)^{5+}$	1.86	31.48
1260.48476	5	$(z_{55}+2Ac)^{5+\bullet}$	0.01	22.32
1270.19364	2	$(z_{22}+Ac)^{2+}$	1.86	20.28
1304.69863	4	$(c_{44}+6Ac)^{4+}$	-1.60	6.19
1315.90281	5	$(c_{57}+5Ac)^{5+\bullet}$	0.27	22.43
1320.69952	5	$(y_{57}+4Ac)^{5+}$	-2.60	5.14
1330.70865	5	$(c_{58}+4Ac)^{5+}$	1.02	7.69
1339.11385	5	$(c_{58}+5Ac)^{5+}$	3.32	33.09
1352.47988	4	$(c_{47}+4Ac)^{4+}$	1.99	4.45
1362.78843	1	$(a_{12}+Ac)^{+}$	5.18	3.75
1371.72261	5	$(c_{59}+5Ac)^{5+}$	0.39	20.42
1384.25185	4	$(c_{48}+4Ac)^{4+\bullet}$	2.08	30.92
1395.25330	4	$(c_{49}+2Ac)^{4+\bullet}$	-3.60	21.24
1405.42234	6	$(c_{72}+6Ac)^{6+\bullet}$	3.58	17.73
1405.59051	6	$(c_{72}+6Ac)^{6+}$	3.73	49.19
1405.75836	2	$(y_{24}+2Ac)^{2+}$	-6.30	11.72
1424.43514	6	$(c_{73}+6Ac)^{6+}$	1.77	30.34
1424.60227	6	$[M+6H -H_2O]^{6+}$	-4.23	25.54
1459.95463	6	$(c_{75}+6Ac)^{6+}$	1.09	21.68
1467.12467	6	$[M+6H+6Ac-NH_3]^{6+\bullet\bullet}$	3.99	88.64
1487.55577	4	$(c_{51}+5Ac)^{4+}$	3.96	117.28

Average mass accuracy: 2.25 ppm (standard deviation: 1.58 ppm)

Table S9. Peak assignments for the $[M+7H+4Ac]^{7+}$ fragment ion scan (*m/z* 1249).

m/z ratio	*z*	name	Mass accuracy (ppm)	S/N
996.78607	4	$(a_{35}+3Ac)^{4+}$	-0.63	13.42
1056.31680	4	$(b_{37}+3Ac)^{4+}$	-2.69	6.63
1056.98234	5	$(c_{47}+Ac)^{5+}$	5.18	7.15
1241.81222	7	$[M+7H+3Ac]^{7+}$	-1.59	7.53
1247.81254	7	$[M+7H+4Ac]^{7+}$	-2.53	18536.04
1352.40572	3	$(a_{36}+2Ac)^{3+}$	1.02	8.08
1422.56088	5	$(z_{62}+3Ac)^{5+}$	4.01	10.64
1422.75569	5	$(z_{62}+3Ac)^{5+\bullet}$	-0.74	9.19
1431.11141	6	$(z_{75}+4Ac)^{6+}$	2.92	142.51
1436.45372	6	$(c_{74}+4Ac)^{6+}$	5.41	16.96
1445.95186	6	$(c_{75}+4Ac)^{6+}$	1.62	313.93
1448.62107	6	$[M+6H+3Ac]^{6+}$	3.95	643.91
1452.95204	6	$[M+6H+4Ac-NH_3]^{6+\bullet}$	3.25	762.07
1455.78937	6	$[M+6H+4Ac]^{6+\bullet}$	2.95	5697.09
1565.64506	5	$(z_{69}+3Ac)^{5+\bullet}$	1.79	10.59
1585.65630	5	$(z_{70}+3Ac)^{5+}$	3.83	18.77
1585.85685	5	$(z_{70}+3Ac)^{5+\bullet}$	3.19	16.98
1691.52229	5	$(z_{74}+4Ac)^{5+}$	4.01	38.15
1692.32475	5	$(c_{73}+4Ac)^{5+}$	6.67	53.06
1717.13467	5	$(z_{75}+4Ac)^{5+}$	4.34	11.43
1734.74387	5	$(c_{75}+4Ac)^{5+\bullet}$	4.30	15.55
1734.94439	5	$(c_{75}+4Ac)^{5+}$	3.70	44.33
1735.14491	5	$[M+5H+3Ac-NH_3]^{5+\bullet\bullet}$	5.84	46.42
1738.14638	5	$[M+5H+3Ac]^{5+}$	5.42	214.61
1743.54655	5	$[M+5H+4Ac-NH_3]^{5+\bullet\bullet}$	5.54	122.79
1746.95280	5	$[M+5H+4Ac]^{5+\bullet\bullet}$	6.07	835.46

Average mass accuracy: 3.58 ppm (standard deviation: 1.67 ppm)

Table S10. Peak assignments for the $[M+7H+5Ac]^{7+}$ fragment ion scan (*m/z* 1255).

m/z ratio	*z*	name	Mass accuracy (ppm)	S/N
386.22754	1	z_4	0.82	8.48
390.21731	1	c_3	0.91	2.68
588.32981	2	$(a_{10}+2Ac)^{2+}$	2.59	7.43
636.35289	1	c_5	-1.40	12.93
806.45820	1	$(c_6+Ac)^{+}$	-1.38	9.28
859.45268	3	$(c_{22}+3Ac)^{3+}$	-3.60	5.55
867.00267	2	$(z_{15}+Ac)^{2+}$	2.20	14.61
907.50790	1	$(c_{07}+Ac)^{+}$	1.01	7.56
949.52063	1	$(c_{07}+2Ac)^{+}$	3.24	7.47
977.52065	3	$(b_{26}+Ac)^{3+}$	1.47	5.02
990.86382	3	y_{26}	0.45	4.61
1062.09599	2	$(z_{18}+Ac)^{2+}$	0.06	6.96
1079.90462	3	$(z_{28}+Ac)^{3+}$	-3.31	9.38
1181.83906	5	$(c_{51}+4Ac)^{5+}$	-0.16	6.31
1247.81246	7	$[M+7H+4Ac]^{7+}$	-2.60	44.19
1251.24251	7	$[M+7H+5Ac-H_2O]^{7+}$	-1.41	3.12
1251.38665	7	$[M+7H+5Ac-NH_3]^{7+}$	1.44	3.67
1253.81555	7	$[M+7H+5Ac]^{7+}$	-1.33	101474.31
1270.69899	2	$(z_{22}+Ac)^{2+\bullet}$	2.99	22.42
1278.20210	2	$(y_{22}+Ac)^{2+}$	1.14	4.36
1303.70937	3	$(z_{34}+2Ac)^{3+}$	0.74	14.50
1348.76891	1	$(c_{11}+2Ac)^{+}$	2.36	13.08
1396.75461	4	$(z_{48}+3Ac)^{4+}$	3.04	4.02
1397.00530	4	$(z_{48}+3Ac)^{+\bullet}$	2.13	7.45
1400.75854	4	$(y_{48}+3Ac)^{4+}$	2.50	5.39
1414.51443	4	$(z_{49}+3Ac)^{4+}$	3.39	11.95
1414.76512	4	$(z_{49}+3Ac)^{+\bullet}$	2.49	13.08
1422.55959	5	$(z_{62}+3Ac)^{5+}$	3.10	7.57
1422.76014	5	$(z_{62}+3Ac)^{5+\bullet}$	2.39	16.82
1431.27862	6	$(z_{75}+4Ac)^{6+\bullet}$	2.38	60.92
1438.11189	6	$(z_{75}+5Ac)^{6+}$	2.01	201.16
1443.45169	6	$(c_{74}+5Ac)^{6+}$	2.76	199.19
1445.78763	6	$(c_{75}+4Ac)^{6+\bullet}$	4.20	114.36
1455.62205	6	$[M+6H+4Ac]^{6+}$	3.39	5500.03
1462.62373	6	$[M+6H+5Ac]^{6+}$	3.33	9674.97
1485.59791	5	$(z_{65}+3Ac)^{5+}$	4.61	54.35
1485.79970	5	$(z_{65}+3Ac)^{5+\bullet}$	4.76	10.56
1559.08997	4	$(c_{54}+4Ac)^{4+}$	3.44	152.18
1573.85523	2	$(c_{28}+Ac)^{2+}$	0.46	12.85
1574.04559	5	$(z_{69}+4Ac)^{5+\bullet}$	0.77	14.46
1594.06024	5	$(z_{70}+4Ac)^{5+}$	4.95	24.60
1594.26079	5	$(z_{70}+4Ac)^{5+\bullet}$	4.32	69.98
1621.87182	3	$(c_{42}+3Ac)^{3+}$	4.05	19.34
1628.07691	5	$(z_{71}+5Ac)^{5+}$	2.12	40.14

1628.27850	5	$(z_{71}$+5Ac$)^{5+\bullet}$	2.14	30.19
1630.21186	3	$(z_{43}$+Ac$)^{3+\bullet}$	2.91	8.25
1634.12969	4	$(c_{57}$+4Ac$)^{4+\bullet}$	3.72	8.36
1634.38032	4	$(c_{57}$+4Ac$)^{4+}$	2.91	23.42
1647.89503	5	$(z_{72}$+5Ac$)^{5+}$	4.79	22.60
1648.09559	5	$(z_{72}$+5Ac$)^{5+\bullet}$	4.18	19.18
1663.14099	4	$(c_{58}$+4Ac$)^{4+}$	5.23	71.87
1677.90833	5	$(c_{72}$+5Ac$)^{5+\bullet}$	6.64	134.83
1678.10875	5	$(c_{72}$+5Ac$)^{5+}$	5.95	169.08
1691.72656	5	$(z_{74}$+4Ac$)^{5+\bullet}$	5.60	52.45
1692.89784	4	$(z_{59}$+3Ac$)^{4+\bullet}$	1.48	13.36
1699.92295	5	$(z_{74}$+5Ac$)^{5+}$	3.13	117.40
1700.72504	5	$(c_{73}$+5Ac$)^{5+}$	5.57	166.48
1725.73794	5	$(z_{75}$+5Ac$)^{5+\bullet}$	4.08	57.46
1725.93856	5	$(a_{75}$+4Ac$)^{5+}$	2.83	32.70
1731.73613	5	$(c_{74}$+5Ac$)^{5+\bullet}$	1.10	10.50
1731.93719	5	$(c_{74}$+5Ac$)^{5+}$	0.80	12.10
1734.74463	5	$(c_{75}$+4Ac$)^{5+\bullet}$	4.74	66.05
1738.14656	5	[M+5H+3Ac]$^{5+}$	5.52	11.06
1743.34702	5	$(c_{75}$+5Ac$)^{5+}$	3.98	325.24
1746.54839	5	[M+5H+4Ac]5+	5.34	1285.89
1751.95023	5	[M+5H+5Ac-NH_3]$^{5+\bullet\bullet}$	6.41	503.72
1755.15136	5	[M+5H+5Ac]$^{5+\bullet}$	4.91	684.29
1755.35142	5	[M+5H +5Ac]$^{5+\bullet\bullet}$	4.05	1752.71
1760.94188	4	$(c_{62}$+Ac$)^{4+\bullet}$	4.12	49.84
1761.19256	4	$(c_{62}$+Ac$)^{4+}$	3.38	17.07
1788.96969	3	$(c_{47}$+3Ac$)^{3+}$	3.38	18.67
1835.23774	4	$(c_{63}$+5Ac$)^{4+}$	9.18	5.14
1835.48835	4	$(y_{64}$+3Ac$)^{4+}$	4.68	7.03
1867.49562	4	$(z_{65}$+4Ac$)^{4+\bullet}$	2.15	10.99
1889.22311	4	$(c_{65}$+5Ac$)^{4+}$	-8.71	5.59
2097.13604	2	$(z_{36}$+2Ac$)^{2+}$	-1.33	4.13
2097.63740	2	$(z_{36}$+2Ac$)^{2+\bullet}$	-2.55	6.38
2124.91072	4	$(z_{74}$+5Ac$)^{4+\bullet}$	6.38	15.70
2125.41212	4	$(z_{75}$+2Ac$)^{4+\bullet}$	3.87	14.43
2179.18932	4	[M+4H+4Ac-H_2O]$^{4+\bullet\bullet\bullet}$	6.46	61.56
2179.43656	4	[M+4H+4Ac-NH_3]$^{4+\bullet\bullet\bullet}$	7.03	33.13
2189.93335	4	[M+4H+5Ac-NH_3]$^{4+\bullet\bullet\bullet}$	4.32	3.99
2910.56837	2	$(c_{51}$+2Ac$)^{2+\bullet}$	-1.49	5.92
2925.24529	3	[M+3H+5Ac]$^{3+\bullet\bullet\bullet}$	2.40	153.16
2925.57950	3	[M+3H+5Ac]$^{3+\bullet\bullet\bullet\bullet}$	1.80	170.07

Average mass accuracy: 3.23 ppm (standard deviation: 1.89 ppm)

Table S11. Peak assignments for the $[M+7H+5Ac]^{7+}$ fragment ion scan (*m/z* 1260).

m/z ratio	*z*	name	Mass accuracy (ppm)	S/N
386.22752	1	z_4	0.77	10.34
1062.09719	2	$(z_{18}+Ac)^{2+}$	1.19	22.42
1085.24911	3	$(y_{28}+Ac)^{3+}$	1.24	4.99
1092.59564	4	$(c_{39}+Ac)^{4+}$	4.32	50.01
1098.59836	2	z_{19}^{2+}	-5.24	21.65
1102.33864	4	$(a_{39}+3Ac)^{4+}$	-1.99	92.42
1102.84585	4	a_{40}^{4+}	-1.54	327.43
1134.28497	3	$(c_{29}+4Ac)^{3+}$	3.52	5.90
1136.61004	3	$(z_{29}+2Ac)^{3+}$	0.00	3.53
1157.64648	3	$(a_{31}+Ac)^{3+}$	2.02	6.21
1174.84173	5	z_{52}^{5+}	-2.01	4.14
1202.30096	6	$(y_{62}+5Ac)^{+}$	-2.39	4.34
1259.81889	7	$[M+7H+6Ac]^{7+}$	0.13	18543.66
1264.74628	1	c_{11}	1.33	15.06
1270.19757	2	$(z_{22}+Ac)^{2+}$	4.95	33.99
1270.70107	2	$(z_{22}+Ac)^{2+\bullet}$	4.63	23.70
1297.70003	6	$(z_{69}+2Ac)^{6+}$	0.00	12.35
1438.11401	6	$(z_{75}+5Ac)^{6+}$	3.49	45.45
1438.28144	6	$(z_{75}+5Ac)^{6+\bullet}$	3.11	34.68
1450.45428	6	$(c_{74}+6Ac)^{6+}$	3.32	339.24
1455.62446	6	$[M+6H+4Ac]^{6+}$	5.05	8.20
1455.79156	6	$[M+6H+4Ac]^{6+\bullet}$	4.46	8.45
1466.95578	6	$[M+6H+6Ac-NH_3]^{6+\bullet}$	3.37	1554.75
1469.79274	6	$[M+6H+6Ac]^{6+\bullet}$	2.82	16401.47
1479.45676	3	$(z_{38}+2Ac)^{3+}$	-0.04	6.78
1545.86633	1	$(b_{13}+2Ac)^{+}$	-2.97	3.65
1548.57897	4	$(c_{54}+3Ac)^{4+}$	-1.94	7.29
1569.59340	4	$(c_{54}+5Ac)^{4+}$	3.91	271.58
1594.84914	4	$(c_{55}+5Ac)^{4+}$	-0.02	23.63
1644.88943	4	$(c_{57}+5Ac)^{4+}$	6.83	7.89
1662.90009	2	$z_{29}^{2+\bullet}$	-2.81	14.40
1673.64252	4	$(c_{58}+5Ac)^{4+}$	4.54	99.33
1686.50900	5	$(c_{72}+6Ac)^{5+}$	4.82	224.66
1700.12960	5	$(z_{74}+5Ac)^{5+\bullet}$	6.12	42.49
1700.32656	5	$(z_{75}+2Ac)^{5+}$	2.09	20.17
1709.12524	5	$(c_{73}+6Ac)^{5+}$	4.42	224.14
1740.33863	5	$(c_{74}+6Ac)^{5+}$	0.41	22.13
1742.94268	5	$[M+5H+4Ac-H_2O]^{5+}$	3.28	34.42
1743.14353	5	$(c_{75}+5Ac)^{5+\bullet}$	2.87	54.81
1751.75064	5	$(c_{75}+6Ac)^{5+}$	4.82	451.96
1754.95162	5	$[M+5H+5Ac]^{5+}$	5.95	1317.39
1760.35124	5	$[M+5H+6Ac-NH_3]^{5+\bullet\bullet}$	5.75	583.75
1763.75634	5	$[M+5H+6Ac]^{5+\bullet\bullet}$	5.62	2311.76
1803.32157	3	$(c_{48}+Ac)^{3+\bullet}$	1.44	9.74

1845.34447	3	$(a_{49}+2Ac)^{3+}$	3.79	21.39
1860.00925	3	$(c_{49}+2Ac)^{3+\bullet}$	0.31	13.99
1878.00332	4	$(z_{65}+5Ac)^{4+\bullet}$	4.83	36.66
1983.07555	3	$(c_{51}+5Ac)^{3+}$	5.78	24.29
1987.27699	4	$(c_{68}+6Ac)^{4+\bullet}$	-5.49	18.16
2189.69707	4	$[M+4H+5Ac-H_2O]^{4+\bullet\bullet\bullet}$	8.76	109.43
2198.20006	2	$(z_{38}+Ac)^{2+\bullet}$	9.02	10.70
2200.18712	4	$[M+4H+6Ac-NH_3]^{4+\bullet\bullet}$	5.70	6.83
2200.44512	4	$[M+4H+6Ac-NH_3]^{4+\bullet\bullet\bullet}$	8.45	5.94
2518.38484	1	a_{23}	0.46	50.30
2939.24953	3	$[M+3H+6Ac]^{3+\bullet\bullet\bullet}$	2.63	288.68
2939.58762	3	$[M+3H+6Ac]^{3+\bullet\bullet\bullet\bullet}$	3.36	310.82

Average mass accuracy: 3.43 ppm (standard deviation: 2.25 ppm)

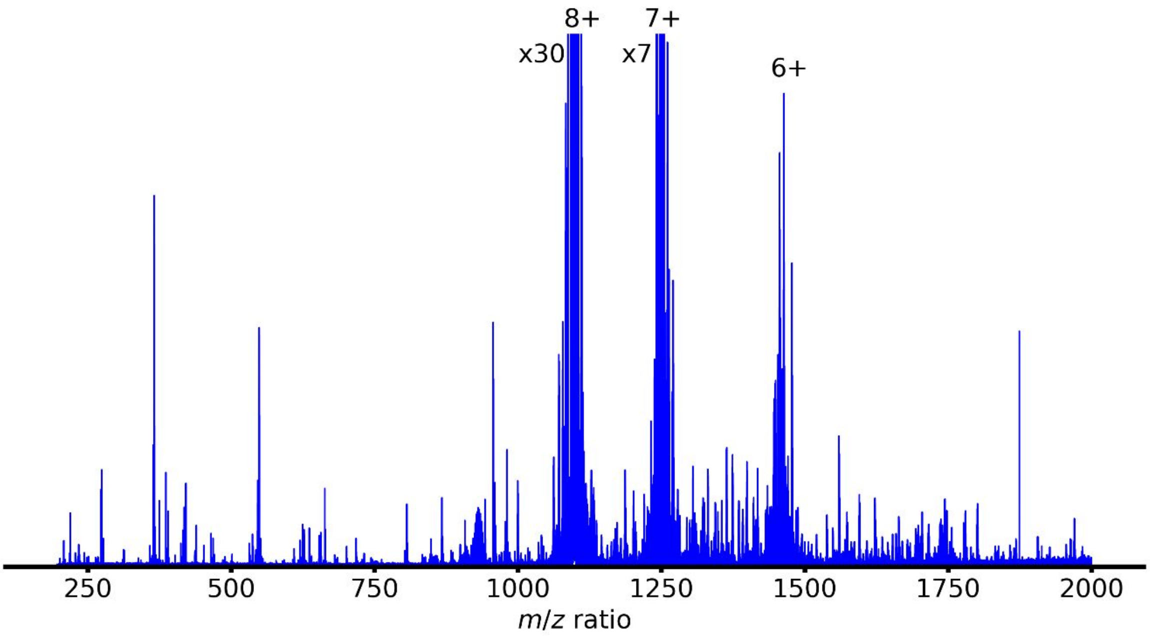

Figure S2. Tandem mass spectrum of acetylated ubiquitin. Quadrupole isolation at m/z 1097 with *m/z* 60 window. 0.5 s accumulation in the collision cell with 5 V collision voltage. ECD fragmentation with 1.3 A current, 5 V lens, 0.7 V bias, 0.04 s irradiation (these parameters are different from the ones used for the acquisition of the 2D mass spectrum due to a cathode regeneration). 2M data points with 937550 Hz Nyquist frequency in 2XR detection (*m/z* 196.51 – 3000). 200 scans accumulated.

Table S12. Peak assignments for the $[M+7H+(1\text{-}6)Ac]^{8+}$ tandem mass spectrum (*m/z* 1097).

m/z ratio	*z*	name	Mass accuracy (ppm)	S/N
273.14355	1	z_3	1.46	13.12
277.13287	1	c_2	-0.07	4.82
386.22745	1	z_4	0.59	16.85
390.21717	1	c_3	0.56	10.60
537.28514	1	c_4	-0.41	6.80
542.32812	1	z_5	-0.39	2.17
609.88184	2	z_{11}^{2+}	0.71	5.09
636.35353	1	c_5	-0.39	8.62
653.39639	2	z_{12}^{2+}	-1.57	8.24
655.41180	1	z_6	-0.91	6.03
717.91930	2	z_{13}^{2+}	0.81	8.10
802.97245	2	$(z_{14}+Ac)^{2+}$	1.21	5.73
806.45963	1	$(c_6+Ac)^{+}$	0.40	14.84
833.12535	3	z_{22}^{3+}	-1.29	4.68
838.46514	3	y_{22}^{3+}	-1.02	4.33
847.12823	3	$(z_{22}+Ac)^{3+}$	-2.02	5.98
848.46914	1	$(c_6+2Ac)^{+}$	-0.86	6.89
867.00005	2	$(z_{15}+Ac)^{+}$	-0.82	10.66
867.56537	1	z_8	0.57	15.88
868.57214	1	$z_8^{\bullet}$	-0.64	6.41
883.58255	1	y_8	-1.19	3.56
884.72309	4	$(z_{31}+2Ac)^{4+}$	-1.02	4.03
899.16277	3	$(z_{23}+Ac)^{3+}$	-0.97	11.71
902.53727	2	z_{16}^{2+}	-0.27	2.59
907.50562	1	$(c_7+Ac)^{+}$	-1.50	10.91
910.98737	4	$(z_{32}+Ac)^{4+}$	-1.19	4.64
918.17024	3	$(z_{24}+Ac)^{3+}$	-0.61	7.87
921.49003	4	$(z_{32}+2Ac)^{4+}$	-1.15	13.69
923.54190	2	$(z_{16}+Ac)^{2+}$	-0.97	8.06
942.50842	3	z_{25}^{2+}	-1.25	31.63
949.51324	5	$(z_{41}+2Ac)^{5+}$	-1.76	4.36
956.51188	3	$(z_{25}+Ac)^{3+}$	-1.30	115.83
959.55816	2	z_{17}^{2+}	-0.86	24.37
961.85236	3	$(y_{25}+Ac)^{3+}$	-0.34	5.54
978.03124	4	$(z_{34}+2Ac)^{4+}$	-1.93	20.51
980.56297	2	$(z_{17}+Ac)^{2+}$	-1.31	48.50
985.52275	3	z_{26}^{3+}	-1.06	6.66
994.55887	2	$(b_{17}+2Ac)^{2+}$	-1.60	6.53
999.52582	3	$(z_{26}+Ac)^{3+}$	-1.50	42.08
1004.62504	1	z_9	1.25	3.04
1012.34490	5	$(z_{44}+2Ac)^{5+}$	-1.80	5.48
1017.05717	4	$(z_{35}+2Ac)^{4+}$	-1.22	10.00
1020.58954	1	$(c_8+Ac)^{+}$	-1.48	3.69
1035.35094	5	$(z_{45}+2Ac)^{5+}$	-1.13	14.51

1037.22000	3	$(z_{27}$+Ac$)^{3+}$	-1.93	5.76
1041.08882	2	z_{18}^{2+}	-1.75	13.03
1043.75259	5	$(z_{45}$+3Ac$)^{5+}$	-1.56	10.66
1060.96194	5	$(z_{46}$+2Ac$)^{5+}$	-1.78	17.13
1062.09411	2	$(z_{18}$+Ac$)^{2+}$	-1.71	37.51
1070.10337	2	$(y_{18}$+Ac$)^{2+}$	-1.79	7.88
1070.95730	8	$[M+8H]^{8+}$	-1.93	121.22
1076.20798	8	$[M+8H+Ac]^{8+}$	-2.52	33.43
1077.83238	4	b_{39}^{4+}	0.69	85.34
1081.46043	8	$[M+8H+2Ac]^{8+}$	-1.46	37.98
1086.71086	8	$[M+8H+3Ac]^{8+}$	-2.27	424.73
1091.96243	8	$[M+8H+4Ac]^{8+}$	-2.03	3173.66
1094.96168	8	$[M+8H+5Ac-H_2O]^{8+}$	-2.71	59.19
1097.21377	8	$[M+8H+5Ac]^{8+}$	-2.00	7698.29
1109.84413	4	$(z_{38}$+2Ac$)^{4+}$	-0.27	74.46
1117.60174	5	$(z_{48}$+3Ac$)^{5+}$	-0.01	12.95
1119.60787	2	$(z_{19}$+Ac$)^{2+}$	-1.36	19.22
1120.80349	5	$(y_{48}$+3Ac$)^{5+}$	-1.79	35.48
1121.63685	1	$(c_{9}$+Ac$)^{+}$	-1.67	4.64
1127.61710	2	$(y_{19}$+Ac$)^{2+}$	-1.47	32.68
1128.76232	6	$(z_{59}$+3Ac$)^{6+}$	-2.09	5.32
1131.80671	5	$(z_{49}$+3Ac$)^{5+}$	-2.17	43.48
1136.60776	3	$(z_{29}$+2Ac$)^{3+}$	-2.00	25.78
1143.63185	3	c_{30}+2Ac$)^{3+\bullet}$	-0.73	5.29
1145.27514	6	$(z_{60}$+3Ac$)^{6+}$	-0.82	17.37
1155.61421	3	$(z_{30}$+2Ac$)^{3+}$	-2.58	11.81
1162.37322	4	$(y_{40}$+2Ac$)^{4+}$	-1.96	14.39
1166.78028	6	$(z_{61}$+3Ac$)^{6+}$	-2.49	14.52
1169.45002	6	$(y_{61}$+3Ac$)^{6+}$	-2.53	14.38
1172.64913	3	$(c_{31}$+Ac$)^{3+}$	-1.85	17.57
1179.29376	3	$(z_{31}$+2Ac$)^{3+}$	-2.10	14.38
1186.65195	3	$(c_{31}$+2Ac$)^{3+}$	-2.41	34.49
1188.13021	6	$(c_{61}$+6Ac$)^{6+}$	3.66	4.94
1188.29733	6	$(c_{62}$+3Ac$)^{6+\bullet}$	1.37	7.21
1190.39309	4	$(z_{42}$+Ac$)^{4+}$	-1.23	4.51
1190.64378	4	$(z_{42}$+Ac$)^{4+\bullet}$	-2.29	3.74
1195.29994	6	$(y_{62}$+4Ac$)^{6+}$	-1.78	7.48
1200.65612	3	c_{31}+3Ac$)^{3+}$	-1.85	7.17
1200.89485	4	$(z_{42}$+2Ac$)^{4+}$	-1.96	19.31
1201.14579	4	$(z_{42}$+2Ac$)^{4+\bullet}$	-2.80	19.52
1204.89851	4	$(y_{42}$+2Ac$)^{4+}$	-2.80	16.08
1205.14037	6	$(y_{63}$+3Ac$)^{6+}$	-1.01	6.82
1210.65712	3	$(c_{32}$+Ac$)^{3+\bullet}$	-0.45	4.55
1210.99134	3	$(c_{32}$+Ac$)^{3+}$	-1.87	3.32
1214.31367	3	$(z_{32}$+Ac$)^{3+}$	-1.52	4.38

1216.39616	4	(c_{42}+3Ac)$^{4+\bullet}$	-2.17	3.97
1216.64682	4	(c_{42}+3Ac)$^{4+}$	-3.24	6.62
1219.66441	2	(z_{21}+Ac)$^{2+}$	-2.49	25.61
1222.65304	4	(z_{43}+Ac)$^{4+}$	-1.77	4.22
1223.80663	7	[M+7H] $^{7+}$	-2.48	14.06
1223.95015	7	[M+7H] $^{7+\bullet}$	-2.85	11.85
1226.80397	7	(z_{75}+4Ac)$^{7+}$	-2.67	30.88
1228.31618	3	(z_{32}+2Ac)$^{3+}$	-2.33	17.60
1229.09096	7	(y_{75}+4Ac)$^{7+}$	-3.80	8.52
1229.80707	7	[M+7H+Ac]$^{7+}$	-3.33	6.17
1230.46247	5	(c_{54}+2Ac)$^{5+\bullet}$	-0.72	4.19
1231.37754	7	(c_{74}+4Ac)$^{7+}$	-4.66	80.80
1232.80717	7	(z_{75}+5Ac)$^{7+}$	-1.28	30.55
1232.95006	7	(z_{75}+5Ac)$^{7+\bullet}$	-2.16	6.71
1233.15425	4	(z_{43}+2Ac)$^{4+}$	-2.91	23.39
1233.38044	7	[M+7H+2Ac-NH_3]$^{7+}$	0.10	7.97
1233.40715	4	(z_{43}+2Ac)$^{4+\bullet}$	-2.14	3.24
1233.52371	7	[M+7H+2Ac-NH_3]$^{7+\bullet}$	-0.47	14.02
1241.81082	7	[M+7H+3Ac]$^{7+}$	-2.71	339.69
1247.81275	7	[M+7H+4Ac]$^{7+}$	-2.37	1786.68
1253.95767	7	[M+7H+5Ac]$^{7+\bullet}$	-2.80	1949.98
1259.95925	7	[M+7H+6Ac]$^{7+\bullet}$	-2.74	253.65
1270.18913	2	(z_{22}+Ac)$^{2+}$	-1.70	74.39
1272.18020	4	(b_{43}+6Ac)$^{4+}$	5.61	8.31
1272.93848	4	(c_{44}+3Ac)$^{4+\bullet}$	-1.85	3.31
1273.18981	4	(c_{44}+3Ac)$^{4+}$	-2.34	10.51
1275.68205	4	(z_{44}+3Ac)$^{4+}$	-1.71	9.85
1275.93273	4	(z_{44}+3Ac)$^{4+\bullet}$	-2.70	14.33
1278.19660	2	(y_{22}+Ac)$^{2+}$	-3.16	23.54
1281.69565	3	(c_{33}+3Ac)$^{3+}$	-2.59	21.52
1290.29246	5	(c_{56}+4Ac)$^{5+}$	-2.35	6.21
1293.93482	4	(z_{45}+2Ac)$^{4+}$	-2.70	9.93
1294.18551	4	(z_{45}+2Ac)$^{4+\bullet}$	-3.68	12.99
1296.03485	3	$b_{35}{}^{3+}$	0.47	5.96
1299.09583	5	(c_{57}+3Ac)$^{5+\bullet}$	-1.84	31.32
1304.68800	4	(z_{45}+3Ac)$^{4+\bullet}$	-3.77	34.48
1306.75137	1	(c_{11}+Ac)$^{4+}$	-2.89	4.17
1306.79194	1	$z_{12}{}^{+\bullet}$	-2.63	3.48
1307.69898	5	(c_{57}+4Ac)$^{5+}$	-2.23	29.88
1311.35627	6	(c_{68}+4Ac)$^{6+}$	-1.93	15.79
1318.35550	6	(c_{68}+5Ac)$^{6+}$	-3.85	15.28
1321.53880	6	(z_{70}+3Ac)$^{6+}$	-3.23	28.15
1322.10119	5	(c_{58}+3Ac)$^{5+\bullet}$	-1.83	33.70
1324.37558	3	(c_{34}+3Ac)$^{3+\bullet}$	-1.25	24.78

1328.54049	6	$(z_{70}$+4Ac$)^{6+}$	-3.27	10.11
1329.37864	3	$(c_{35}$+2Ac$)^{3+\bullet}$	-1.67	4.80
1329.71322	3	$(c_{35}$+2Ac$)^{3+}$	-2.69	6.52
1330.70344	5	$(c_{58}$+4Ac$)^{5+}$	-2.89	51.60
1331.22054	4	$(c_{47}$+2Ac$)^{4+\bullet}$	0.44	6.60
1336.45251	4	$(z_{46}$+3Ac$)^{4+}$	-2.32	7.96
1336.70320	4	$(z_{46}$+3Ac$)^{4+\bullet}$	-3.26	6.26
1341.97091	4	$(c_{47}$+3Ac$)^{4+}$	-2.71	17.36
1343.71733	3	$(c_{35}$+3Ac$)^{3+}$	-2.23	30.53
1346.10963	5	$(z_{59}$+2Ac$)^{5+\bullet}$	-4.45	8.13
1348.76288	1	$(c_{11}$+2Ac$)^{+}$	-2.10	15.48
1349.88945	6	$(z_{71}$+4Ac$)^{6+}$	-3.36	6.42
1354.31262	5	$(z_{59}$+3Ac$)^{5+}$	-2.61	18.94
1354.91410	5	$(c_{59}$+3Ac$)^{5+}$	-2.76	26.63
1356.07315	3	$(z_{35}$+2Ac$)^{3+\bullet}$	-3.62	5.15
1356.89133	6	$(z_{71}$+5Ac$)^{6+}$	-3.26	12.69
1359.56206	6	$(y_{71}$+5Ac$)^{6+}$	-2.56	7.01
1363.31613	5	$(c_{59}$+4Ac$)^{5+}$	-2.81	51.86
1371.71775	5	$(c_{59}$+5Ac$)^{5+}$	-3.15	10.32
1373.74376	4	$(c_{48}$+3Ac$)^{4+\bullet}$	-1.87	50.01
1384.24571	4	$(c_{48}$+4Ac$)^{4+\bullet}$	-2.36	18.51
1384.49579	4	$(c_{48}$+4Ac$)^{4+}$	-3.71	21.81
1390.90982	6	$(z_{73}$+4Ac$)^{6+}$	-5.01	24.60
1391.57776	6	$(c_{72}$+4Ac$)^{6+}$	-2.87	31.54
1398.41210	6	$(c_{72}$+5Ac$)^{6+\bullet}$	-2.46	30.44
1398.57891	6	$(c_{72}$+5Ac$)^{6+}$	-3.29	25.72
1403.25661	6	$(c_{73}$+3Ac$)^{6+\bullet}$	-1.97	7.87
1404.00328	4	$(z_{49}$+2Ac$)^{4+}$	-2.65	7.48
1405.57999	6	$(c_{72}$+6Ac$)^{6+}$	-3.76	8.97
1409.75909	6	$(z_{74}$+4Ac$)^{6+}$	-3.58	36.79
1410.42496	6	$(c_{73}$+4Ac$)^{6+}$	-2.93	38.14
1414.75673	4	$(z_{49}$+3Ac$)^{4+\bullet}$	-3.44	25.96
1416.92499	6	$(z_{74}$+5Ac$)^{6+\bullet}$	-6.27	6.91
1417.42566	6	$(c_{73}$+5Ac$)^{6+}$	-3.66	53.25
1422.55200	5	$(b_{62}$+3Ac$)^{5+}$	1.66	5.85
1422.75248	5	$(z_{62}$+3Ac$)^{5+\bullet}$	-3.00	9.36
1423.93086	6	$(z_{74}$+6Ac$)^{6+\bullet}$	-3.36	4.98
1424.26383	6	$(c_{73}$+6Ac$)^{6+\bullet}$	-0.57	3.48
1424.43019	6	$(c_{73}$+6Ac$)^{6+}$	-1.71	2.17
1425.75311	5	$(c_{62}$+3Ac$)^{5+\bullet}$	-0.20	9.50
1429.27149	6	$(c_{74}$+3Ac$)^{6+\bullet}$	-3.31	11.13
1455.61353	6	$[M+6H+4Ac]^{6+}$	-2.46	269.03
1469.95154	6	$[M+6H+6Ac]^{6+\bullet\bullet}$	-3.42	69.22
1477.04298	4	$(c_{51}$+4Ac$)^{4+}$	-2.88	180.29
1484.45476	3	$(c_{39}$+3Ac$)^{3+}$	-2.97	31.20
1487.54525	4	$(c_{51}$+5Ac$)^{4+}$	-3.11	40.89

1491.04220	4	$(b_{52}+3Ac)^{4+}$	-1.67	7.23
1492.29571	4	$(z_{51}+5Ac)^{4+}$	-6.74	3.78
1494.18198	5	$(c_{64}+5Ac)^{5+}$	-3.15	26.61
1498.78263	2	$(z_{26}+Ac)^{2+}$	-3.14	5.06
1499.28455	2	$(z_{26}+Ac)^{2+\bullet}$	-4.47	4.47
1505.79794	4	$(c_{52}+4Ac)^{4+}$	-4.01	13.41
1509.30042	4	$(c_{53}+3Ac)^{4+\bullet}$	-2.86	3.87
1509.55108	4	$(c_{53}+3Ac)^{4+}$	-3.72	2.76
1511.40704	5	$(z_{66}+3Ac)^{5+\bullet}$	-3.03	13.76
1514.20661	5	$(z_{67}+2Ac)^{5+}$	-3.71	5.31
1520.05507	4	$(c_{53}+4Ac)^{4+}$	-2.81	21.42
1522.60912	5	$(z_{67}+3Ac)^{5+}$	-3.43	3.38
1522.80967	5	$(z_{67}+3Ac)^{5+\bullet}$	-4.10	3.14
1527.14029	3	$(c_{40}+3Ac)^{3+}$	-3.32	8.47
1536.82813	2	$(a_{27}+2Ac)^{2+}$	-1.54	3.81
1537.82994	2	$(c_{27}+Ac)^{2+\bullet}$	-1.36	7.13
1538.33090	2	$(c_{27}+Ac)^{2+}$	-3.28	17.91
1548.32585	4	$(c_{54}+3Ac)^{4+\bullet}$	-2.69	22.65
1551.34196	2	$(a_{28}+Ac)^{2+}$	-1.17	6.79
1555.48912	3	$(c_{41}+2Ac)^{3+\bullet}$	-1.91	7.81
1559.08137	4	$(c_{54}+4Ac)^{4+}$	-2.08	39.03
1559.33536	2	$(c_{27}+2Ac)^{2+}$	-3.77	30.94
1562.89441	1	$(c_{13}+2Ac)^{+}$	-1.96	2.74
1565.63792	5	$(z_{69}+3Ac)^{5+\bullet}$	-2.77	9.45
1569.82501	3	$(c_{41}+3Ac)^{3+}$	-4.17	12.47
1573.35041	2	$(c_{28}+Ac)^{2+\bullet}$	-0.11	20.28
1573.42572	5	$(c_{68}+4Ac)^{5+}$	-2.16	10.30
1573.59379	4	$(c_{55}+3Ac)^{4+\bullet}$	1.18	3.53
1574.04502	5	$(z_{69}+4Ac)^{5+\bullet}$	0.41	4.27
1577.44339	5	$(z_{70}+2Ac)^{5+\bullet}$	-3.99	7.25
1580.33994	2	$(c_{27}+3Ac)^{2+}$	-4.16	9.22
1581.82886	5	$(c_{68}+5Ac)^{5+}$	-1.50	6.87
1582.18281	3	$(z_{41}+2Ac)^{3+\bullet}$	-4.10	7.13
1585.84608	5	$(z_{70}+3Ac)^{5+\bullet}$	-3.60	17.52
1594.85502	2	$(c_{28}+2Ac)^{2+}$	-2.99	32.51
1601.19051	3	$(z_{42}+2Ac)^{3+\bullet}$	-3.70	8.60
1607.85692	3	$(c_{42}+2Ac)^{3+}$	-3.00	17.27
1615.35888	2	$(c_{28}+3Ac)^{2+\bullet}$	-1.41	6.87
1621.85913	3	$(c_{42}+3Ac)^{3+}$	-3.78	30.90
1621.93049	1	$(c_{14}+Ac)^{+}$	-2.52	9.55
1623.61708	4	$(c_{57}+3Ac)^{4+\bullet}$	-2.39	14.33
1627.36835	2	$(y_{28}+Ac)^{2+}$	0.20	3.82
1634.36881	4	$(c_{57}+4Ac)^{4+}$	-4.13	15.92
1637.39453	2	$(c_{29}+Ac)^{2+\bullet}$	-2.16	5.34
1639.68092	5	$(z_{72}+4Ac)^{5+\bullet}$	-3.45	6.81
1641.87088	4	$(c_{58}+2Ac)^{4+\bullet}$	-2.54	5.07

1644.20382	3	(z_{43}+2Ac)$^{3+\bullet}$	-4.15	15.20
1652.37420	4	(c_{58}+3Ac)$^{4+\bullet}$	-2.11	24.00
1658.90078	2	(c_{29}+2Ac)$^{2+}$	-3.91	17.14
1662.87644	4	(c_{58}+4Ac)$^{4+\bullet}$	-2.34	14.24
1663.94011	1	(c_{14}+2Ac)$^{+}$	-3.02	12.45
1669.49082	5	(c_{72}+4Ac)$^{5+\bullet}$	-2.55	23.25
1677.89420	5	(c_{72}+5Ac)$^{5+\bullet}$	-1.79	15.32
1679.90782	2	(c_{29}+3Ac)$^{2+}$	-2.81	6.81
1683.24322	3	(c_{44}+2Ac)$^{3+}$	-4.69	10.63
1691.70982	5	(z_{74}+4Ac)$^{5+\bullet}$	-4.29	5.17
1692.10938	5	(c_{73}+4Ac)$^{5+\bullet}$	-1.49	6.53
1693.13841	4	(c_{59}+3Ac)$^{4+\bullet}$	-3.02	17.77
1693.93707	2	(c_{30}+Ac)$^{2+\bullet}$	-1.79	4.14
1694.43834	2	(c_{30}+Ac)$^{2+}$	-3.35	3.00
1697.24710	3	$z_{45}{}^{3+\bullet}$	1.69	18.16
1700.50860	5	(c_{73}+5Ac)$^{5+\bullet}$	-3.18	10.39
1700.90408	3	(z_{44}+3Ac)$^{3+\bullet}$	-4.94	7.17
1703.64059	4	(c_{59}+4Ac)$^{4+\bullet}$	-3.27	27.47
1710.91688	3	(z_{45}+Ac)$^{3+}$	2.96	3.58
1711.25114	3	(z_{45}+Ac)$^{3+\bullet}$	1.98	2.72
1714.14343	4	(c_{59}+5Ac)$^{4+\bullet}$	-3.14	14.60
1714.94149	2	(c_{30}+2Ac)$^{2+\bullet}$	-2.27	8.82
1715.44259	2	(c_{30}+2Ac)$^{2+}$	-3.91	12.93
1717.32257	5	(z_{75}+4Ac)$^{5+\bullet}$	-3.63	8.12
1724.90862	3	(z_{45}+2Ac)$^{3+}$	-3.89	7.12
1725.72170	5	(z_{75}+5Ac)$^{5+\bullet}$	-5.34	6.35
1726.32213	5	(c_{75}+3Ac)$^{5+\bullet}$	-7.05	6.93
1731.72640	5	(c_{74}+5Ac)$^{5+\bullet}$	-4.52	12.41
1732.00747	1	(a_{15}+2Ac)$^{+}$	-0.15	7.58
1734.01255	1	(c_{15}+Ac)$^{+\bullet}$	1.00	4.11
1734.72675	5	(c_{75}+4Ac)$^{5+\bullet}$	-5.57	21.35
1735.32878	5	[M+5H+3Ac-NH_3]$^{+}$ •••	-4.36	8.30
1736.44877	2	(c_{30}+3Ac)$^{2+}$	-3.35	19.62
1738.33084	5	[M+5H+3Ac]$^{5+\bullet}$	-4.42	21.56
1739.24679	3	(z_{45}+3Ac)$^{3+\bullet}$	-4.61	23.32
1743.52740	5	[M+5H+4Ac-NH_3]$^{5+\bullet\bullet}$	-5.44	42.05
1746.73437	5	[M+5H+4Ac]$^{5+\bullet}$	-3.59	39.67
1751.93292	5	[M+5H+5Ac-NH_3]$^{5+\bullet\bullet}$	-3.47	8.52
1752.13345	5	[M+5H+5Ac-NH_3]$^{5+\bullet\bullet\bullet}$	-4.06	9.70
1755.13569	5	[M+5H+5Ac]$^{5+\bullet}$	-4.02	6.13
1755.33622	5	[M+5H+5Ac]$^{5+\bullet\bullet}$	-4.61	11.91
1755.53683	5	[M+5H+5Ac]$^{5+\bullet\bullet\bullet}$	-5.15	6.41

1758.46658	2	$(c_{31}$+Ac$)^{2+}$	-3.82	7.31
1760.42496	4	$(z61$+4Ac$)^{4+\bullet}$	-0.44	7.88
1777.02307	1	$(c_{15}$+2Ac$)^{+}$	-3.45	17.18
1779.47234	2	$(c_{31}$+2Ac$)^{2+}$	-3.51	18.40
1788.62230	3	$(c_{47}$+3Ac$)^{3+\bullet}$	-3.01	10.75
1800.47793	2	$(c_{31}$+3Ac$)^{2+}$	-3.30	35.82
1831.32147	3	$(c_{48}$+3Ac$)^{3+\bullet}$	-2.48	13.62
1836.48691	2	$(c_{32}$+2Ac$)^{2+\bullet}$	-0.68	3.36
1836.98820	2	$(c_{32}$+2Ac$)^{2+}$	-2.10	6.25
1845.99056	3	$(c_{49}$+Ac$)^{3+\bullet}$	-7.91	6.48
1856.72433	4	$(c_{64}$+4Ac$)^{4+\bullet}$	-1.41	14.47
1864.05353	1	$(c_{16}$+Ac$)^{+}$	-4.13	6.45
1867.22015	4	$(c_{64}$+5Ac$)^{4+\bullet}$	-5.05	13.39
1867.97232	4	$(c_{65}$+3Ac$)^{4+\bullet}$	-5.35	12.05
1906.06354	1	$(c_{16}$+2Ac$)^{+}$	-4.34	12.42
1922.03963	2	$(c_{33}$+3Ac$)^{2+}$	-2.70	6.27
1925.70430	3	$(c_{50}$+4Ac$)^{3+\bullet}$	-3.18	6.81
1926.70695	3	$(c_{51}$+Ac$)^{3+\bullet}$	-3.68	4.64
1954.71532	3	$(c_{51}$+3Ac$)^{3+\bullet}$	-2.95	13.65
1963.12109	1	$(c_{17}$+Ac$)^{+}$	-4.36	11.16
1968.71700	3	$(c_{51}$+4Ac$)^{3+\bullet}$	-3.86	24.40
1970.05592	3	$c_{53}{}^{3+\bullet}$	-2.07	15.13
1982.72033	3	$(c_{51}$+5Ac$)^{3+\bullet}$	-3.93	12.55
2005.13283	1	$(c_{17}$+2Ac$)^{+}$	-3.68	18.76
2026.06752	3	$(c_{53}$+4Ac$)^{3+\bullet}$	-3.24	8.91
2432.28014	1	$(c_{21}$+2Ac$)^{+\bullet}$	-2.70	5.37

Average mass accuracy: 2.49 ppm (standard deviation: 1.34 ppm)

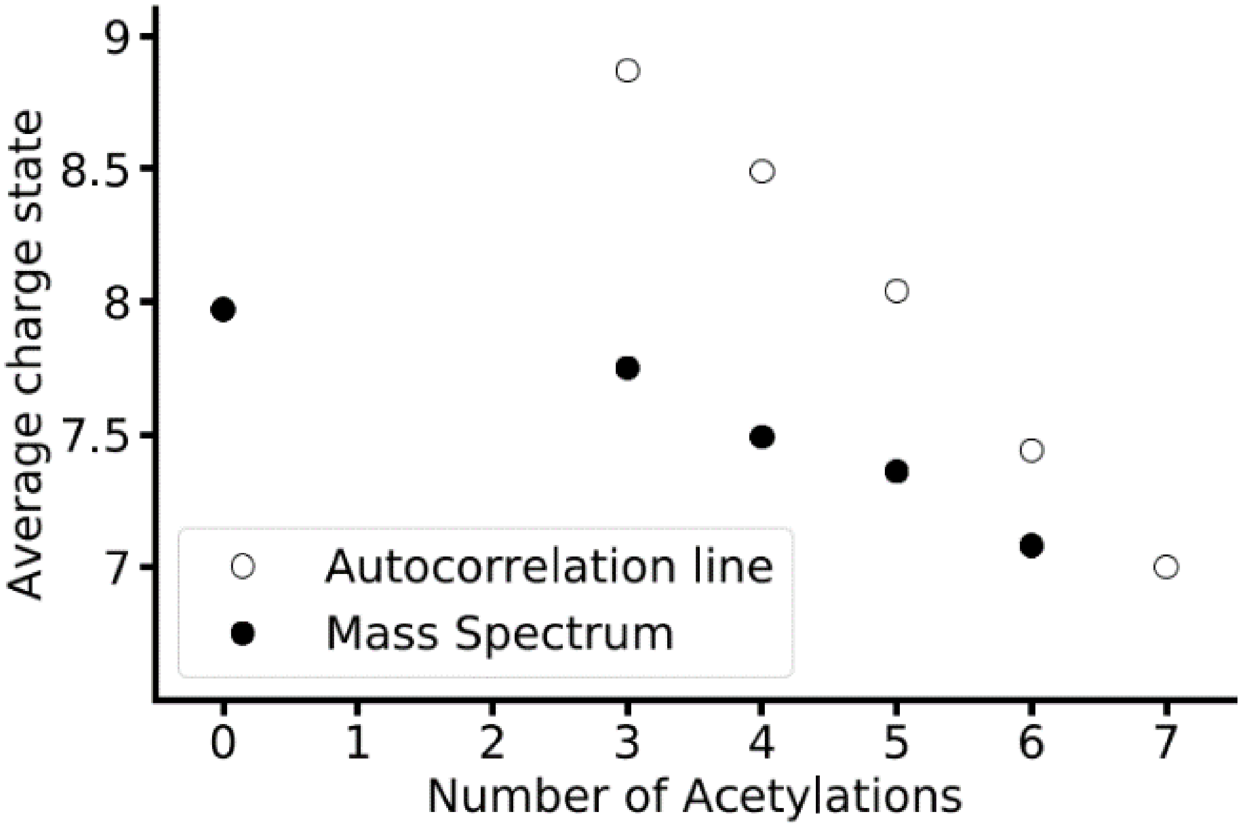

Figure S3. Average charge state vs. number of acetylations calculated from the peak intensities in the autocorrelation line of the 2D mass spectrum and the mass spectrum (all measurable data points are plotted, unmodified ubiquitin was not detected in the 2D mass spectrum).
